# A Circuit of Mechanically Regulated Transcription Factors Balances Regenerative and Fibrotic Memory of Mesenchymal Stromal Cells

**DOI:** 10.1002/advs.202522056

**Published:** 2026-04-22

**Authors:** Fereshteh Sadat Younesi, Andrew E. Miller, Li Diao, Xinying Guo, Natalie Andonian, Elham Karimizadeh, Thomas H. Barker, Boris Hinz

**Affiliations:** ^1^ Laboratory of Tissue Repair and Regeneration Keenan Research Centre For Biomedical Science of the St. Michael's Hospital Ontario Canada; ^2^ Faculty of Dentistry University of Toronto Ontario Canada; ^3^ Department of Biomedical Engineering University of Virginia Charlottesville Virginia USA

**Keywords:** chromatin accessibility, epigenetics, GATA6, HOX family, mechanical memory, myofibroblast, regenerative gene expression, SALL1, scarless wound healing

## Abstract

Therapeutic mesenchymal stromal cells (MSCs) promote healing in severe injuries like skin burns. However, expansion on stiff culture surfaces activates MSCs into scar‐promoting myofibroblasts. We previously introduced ‘mechanical memory’ to describe how MSCs primed on scar‐stiff surfaces retain myofibroblast traits even after switching to softer, skin‐like surfaces. Now, we identify mechanisms and factors that suppress myofibroblast activation during priming in soft cultures. These ‘soft memory’ factors are poised to preserve MSC regenerative features while preventing fibrogenesis. Mechanically primed MSCs were compared via RNA‐ and ATAC‐sequencing to co‐analyze gene transcription and chromatin accessibility. Highly accessible promoters of genes upregulated after soft priming, which retained this pattern after transitioning to stiff surfaces, were enriched for HOXA11 transcription factor binding motifs. Knocking down HOXA11 increased osteogenic gene expression in soft‐primed MSCs and reduced anti‐fibrotic factors, including the transcription factor SALL1, which suppresses pro‐fibrotic genes like *Postn*, *Col8a1*, *Grem2*, *Thbs1*, *Thbs2*, and *Gata6*. We identify GATA6 as a keeper of stiff‐induced myofibroblast memory after switching to soft surfaces. Manipulating the SALL1‐GATA6 circuit yielded therapeutic MSCs that suppressed fibrosis in a hypertrophic skin‐scarring animal model. Therefore, controlling myofibroblast memory could improve MSC‐based organ repair therapies.

## Graphical abstract

Producing MSCs on rigid culture substrates induces a scar‐making phenotype, jeapordizing therapeutic success. ‘Tissue‐soft’ surfaces prevent MSC fibrogenesis and preserve regenerative traits. An epigenetic network, driven by HOXA11 and SALL1, maintains ‘soft memory’ by keeping chromatin open in relaxed MSCs, promoting anti‐fibrotic programs. GATA6, instead, maintains ‘stiff memory’. Modulating this epigenetic regulation enables designing MSCs with desired healing functions for tissue repair.

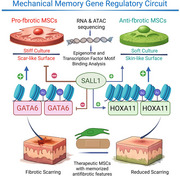

## 1 Introduction

Mesenchymal stromal cells (MSCs) are promising therapeutic agents for accelerating the healing of severe injuries such as burns, while also helping to reduce tissue fibrosis that develops when the body's inherent repair abilities are overwhelmed [1, 2, 3]. The initially low numbers of donor‐derived MSCs must be expanded in cell culture to achieve the billions of cells needed for multiple transplants. However, this expansion often causes MSCs to lose their regenerative potential [4, 5]. This loss is partly due to phenotypic changes triggered by contact with typically stiff culture surfaces, such as the polystyrene used in bioreactor microcarrier beads, flasks, and dishes. A dominant fate driven by mechanical stress, with significant clinical consequences, is the activation of MSCs into myofibroblasts (MF) [6, 7, 8]. MFs mainly support tissue healing by producing a collagen‐rich extracellular matrix (ECM), which is then remodeled into a supportive scar through contractile forces generated by α‐smooth muscle actin (α‐SMA) stress fibers [9]. While transient MFs are beneficial, persistent MFs continue to accumulate stiff, fibrotic scar tissue, further promoting MF activation [10]. The high‐stress environment of injured and fibrotic tissues causes endogenous MF accumulation and drives fibrogenesis in transplanted MSCs, reducing their regenerative capacity [4, 11].

We propose using the 3‐week culture expansion period to produce MSCs that preserve their regenerative capabilities and develop resistance to the fibrotic pressure exerted by recipient tissues. We have previously demonstrated that continuous growth on skin‐soft surfaces (‘priming’) prevents MF activation of MSCs even after transitioning to a new, stiff mechanical environment—a persistent behavior we named ‘mechanical memory’ [12, 13]. Grafting soft‐primed MSCs onto mechanically stressed rodent skin wounds reduced fibrotic features and supported normal healing compared to transplanting conventionally stiff‐expanded MSCs [13]. While we and others have identified molecular factors that convert transient into persistent MF activation during priming on scar‐stiff surfaces (‘stiff memory’) [12, 13, 14, 15, 16], whether specific mechanisms and factors control ‘soft memory’ remains unclear. This study aims to understand how soft memory is established and maintained to suppress persistent mechanical MSC‐to‐MF activation.

Extracellular mechanical cues directly influence chromatin state through force transmission from transmembrane integrins via cytoskeletal fibers to the nuclear envelope [9, 17, 18, 19]. Forces exerted on the outer nuclear envelope are transmitted to the DNA via the linker of nucleoskeleton and cytoskeleton (LINC) complex, which connects to the inner nuclear lamina [20, 21, 22]. Through this axis, extracellular mechanical stress generally causes chromatin to open and increases gene accessibility for transcriptional regulation [15, 23, 24, 25]. Few studies have conceptually linked chromatin accessibility to soft memory [26], but the mechanisms remain largely elusive. We propose that the growth of MSCs on soft ECM also modulates DNA accessibility for specific transcription factors (TFs), which enhances the expression of anti‐fibrotic genes while suppressing the transcription of pro‐fibrotic genes. We explore how mechanical forces interact with transcriptional regulatory networks as a mechanism of soft memory that maintains regenerative capacity and inhibits the MF traits of MSCs.

Using assay for transposase‐accessible chromatin (ATAC) with sequencing (seq), we reveal that 3‐week priming of MSCs on soft surfaces not only significantly increases the accessibility of regulatory regions for 137 genes but also preserves the open chromatin state for 28 genes after transfer to a stiff environment for another 2 weeks. Among the genes with memorized open chromatin following soft priming, 14 exhibit increased transcription levels in RNA‐seq. Among these, we identified homeobox A11 (HOXA11) as an inducer TF for spalt‐like transcription factor 1 (SALL1), which displays similar accessibility and expression patterns as HOXA11. SALL1 is essential for the maintained inhibition of MF fate of MSCs by repressing pro‐fibrotic and pro‐osteogenic genes, including the TF GATA‐binding factor 6 (GATA6). GATA6, in turn, helps sustain stiff MF memory of MSCs, possibly acting as a pioneer TF that preserves stiffness‐induced accessible chromatin even after switching to soft surfaces. Knocking down (KD) SALL1 and GATA6 in MSCs and applying them in an animal model of skin hypertrophic scarring demonstrates the potential of memory‐manipulated MSCs for therapy. Adjusting MSC expansion conditions and/or the gene regulatory circuits that control mechanical memory can generate therapeutic MSCs that resist undesired MF activation in stiff, scar‐forming host environments.

## 2 Results

### 2.1 MSC Culture Substrate Stiffness Controls Chromatin Access and Pro‐ Versus Anti‐Fibrotic Gene Expression

**FIGURE 1:**
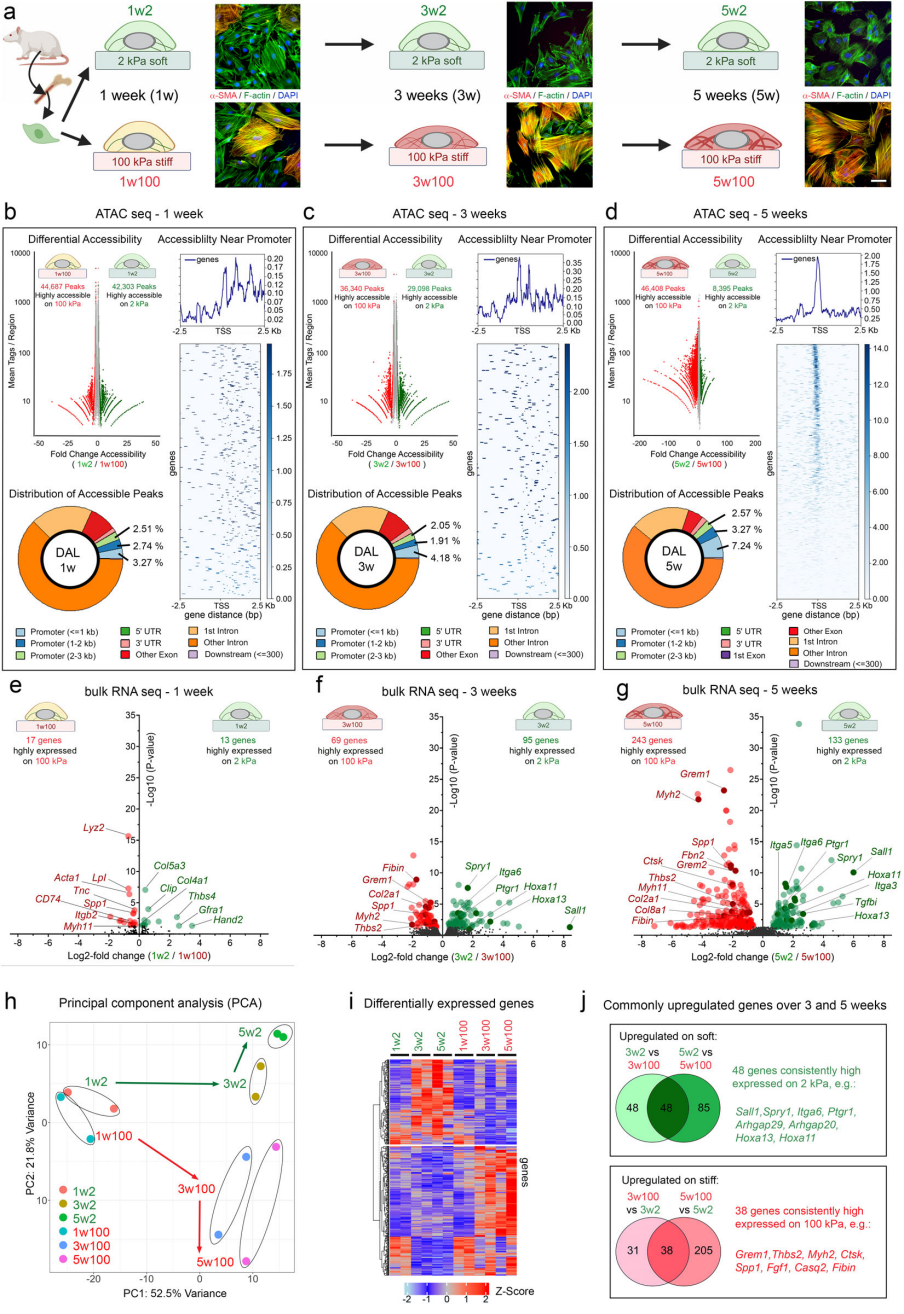
DNA accessibility and transcription landscapes of MSCs in soft and stiff environments. (a) MSCs were isolated from rat bone marrow directly onto gelatin‐coated (10 µg/cm^2^) soft (2 kPa =’2’) and stiff (100 kPa = ’100’) silicone surfaces and mechanically primed for 1 week (1w), 3 weeks (3w), and 5 weeks (5w), with one passage per week. For instance, ‘3w100’ denotes 3 weeks priming on 100 kPa surfaces. Mechanical MF activation is indicated by α‐smooth muscle actin (α‐SMA, red) expression in stress fibers (F‐actin, green). Scale bar: 20 µm. After priming for (b) 1 week, (c) 3 weeks, and (d) 5 weeks, MSC samples were collected to perform ATAC‐seq. Significantly differentially accessible loci (DAL) (*p* ≤ 0.05) between soft‐primed (1w2, 3w2, 5w2) and stiff‐primed MSCs (1w100, 3w100, 5w100) are shown in MA plots (top left in boxes). In MA plots, genes with higher accessibility are shown in green for soft‐primed MSCs and in red for stiff‐primed MSCs. The relative distribution of DAL (*p* < 1e‐4) across genomic regions was determined and presented in pie charts. Heatmaps show ATAC signal density within ±2.5 kb of gene transcription start sites (TSS), with each row representing one single gene. Histograms on top of the heatmaps show the average frequency of accessible loci as a function of distance from the TSS. The same cultures were subjected to bulk RNA‐seq, and differentially expressed genes (DEGs) are shown in volcano plots after (e) 1 week, (f) 3 weeks, and (g) 5 weeks of priming. Significantly DEGs (*p* < 0.05, log‐fold change of >0) are shown in color as described for DAL. (h) Principal component analysis (PCA) of variance‐stabilized expression data was performed using weighted genes from RNA‐seq to cluster MSC transcription profiles by priming time and stiffness (PC1 = 21.8%, PC2 = 52.5%). (i) Z‐scores of RNA‐seq normalized gene expression counts of mechanically primed MSCs are shown in a heatmap, with high expression levels shown in red and low expression in blue. (j) To discover genes consistently upregulated over either soft (green) or stiff priming (red), Venn diagrams were created with significant DEGs after 3 weeks (f) and 5 weeks (g) of MSC priming. Examples of the top overlapping genes are listed. All ATAC‐seq data are shown for two biological replicates (MSCs from two different rats), and statistics for bulk RNA seq were calculated from 3 biological replicates (N = 3), except for 5‐week priming (N = 2).

To explore the long‐term effects of mechanical environment on MSC‐to‐MF activation, we used silicone culture substrates adjusted to the elastic moduli (E) of healthy and diseased tissues [13, 27]. MSCs were directly isolated from rat bone marrow onto culture surfaces with E = 2 kPa to replicate the softness of healthy skin or E = 100 kPa to match the stiffness of fibrotic scar tissue (Figure 1a) [28, 29]. MSCs were then primed for 1 week (1w), 3 weeks (3w), and 5 weeks (5w) on either 2 kPa (‘2’) soft (1w2, 3w2, 5w2) or 100 kPa (‘100’) stiff surfaces (1w100, 3w100, 5w100), with trypsinization and passaging on to fresh culture plates every week. Consistent with our previous research, the percentage of MSCs expressing the MF activation marker α‐SMA increased over time on stiff surfaces but remained low on soft surfaces (Figure 1a); cell proliferation rates were not significantly different on stiff and soft surfaces [13, 30, 31].

Exposure to mechanical stress for a few days or less has been shown to induce chromatin remodeling, affecting gene expression and MSC phenotypes [15, 24, 32]. To understand how physical cues regulate chromatin accessibility in genomic regulatory elements over several weeks of MSC culture, we performed ATAC‐seq on isolated nuclei [33]. The cumulative number of differentially accessible loci (DAL) in MSCs grown on soft versus stiff surfaces, and vice versa, decreased over the priming period (1w: 86 990 DAL; 3w: 65 438 DAL; 5w: 54 803 DAL) (Figure 1b–d, MA plots). In 1‐week primed MSCs, the total number of accessible regions was roughly equal on soft (1w2, green) and stiff (1w100, red) culture surfaces (Figure 1b–d, MA plots, mean tags per region). However, this ratio shifted over time in favor of stiff‐primed MSCs, increasing to 1.25‐fold more accessible regions in stiff‐primed MSCs than in soft‐primed MSCs after 3 weeks (3w100/3w2) and 5.5‐fold after 5 weeks (5w100/5w2). Additionally, the magnitude of DAL detection by ATAC‐seq was up to eightfold higher after 5 weeks compared to 1 and 3 weeks of stiff‐priming (Figure 1b–d, MA plots, ‘‐fold change accessibility’). In contrast, the number of DAL decreased 5‐fold with longer soft‐priming (5w2 versus 1w2), while the magnitude of change was similar (Figure 1b–d). These findings suggest that differences in overall chromatin accessibility become more noticeable with prolonged exposure to a stiff environment. Although fewer in number, a distinct group of gene loci also became more accessible after extended culture on soft surfaces.

DAL that influence MSC function and phenotype are likely located within the promoter regions and/or *cis*‐regulatory enhancer or repressor loci of protein‐coding genes [34]. Therefore, we next examined the distribution of DAL annotations relative to their nearest known gene, such as near the promoter, within the gene body, or in distal intergenic regions (Figure 1b–d). Among all DAL that were more accessible in either soft‐ or stiff‐primed MSCs, the percentage of DAL within 3 kb of a known promoter site increased from 8.5% after 1 week of MSC priming to 13.1% after 5 weeks (Figure 1b–d, pie charts). This suggests that prolonged mechanical priming facilitates chromatin remodeling in regions involved in gene regulation. To analyze the genomic coordinates of promoter‐associated DAL, we evaluated the density distribution of all significant ATAC‐seq peaks around the transcription start site (TSS) of protein‐coding genes (Figure 1b–d, heatmaps). Mechanical priming of MSCs for 5 weeks resulted in a 10‐fold shift in DAL distribution toward the center of the TSS compared to 1‐week priming (Figure 1b–d, graphs above heatmaps), implicating these DAL in activating gene expression during mechanical priming.

Next, to connect mechanically induced DAL with differentially expressed genes (DEGs), we performed RNA‐seq on MSCs harvested from the same culture plates used for ATAC‐seq. Over 5 weeks of priming on soft or stiff surfaces, the total number of DEGs increased by 12.5‐fold (1w: 30 DEGs; 3w: 164 DEGs; 5w: 376 DEGs) (Figure 1e–g, volcano plots, all data points above a significance level of *p* ≤ 0.05). This increase aligned with the greater accumulation of ATAC‐seq peaks around the TSS of protein‐coding genes throughout priming (Figure 1b–d), supporting the idea that accessible gene loci are generally more amenable to transcription. Similarly, the number of genes upregulated in soft‐primed MSCs increased 10‐fold (Figure 1e–g, volcano plots, green data points) and 14‐fold in stiff‐primed MSCs (Figure 1e–g, volcano plots, red data points). After 5 weeks, the number of significantly upregulated genes was twice as high in stiff‐primed MSCs compared to soft‐primed ones. The relationship between DEG kinetics and priming time was also captured by principal component analysis (PCA), an unsupervised dimensionality reduction technique (Figure 1h). The representations of the MSC global transcriptomic landscape clustered closely after 1 week but diverged over time with surface stiffness, with PC1 (52.5% of variance) and PC2 (21.8% of variance) capturing these trends (Figure 1h).

We next focused on DEGs under persistent mechanical control. Heatmap displays illustrated that individual stiffness‐dependent DEGs were retained, and in many cases, further accentuated between weeks 3 and 5 of soft and stiff priming (Figure 1i). Among the 48 persistently upregulated DEGs in soft‐primed MSCs (5w2 ≥ 3w2), were genes associated with regenerative and anti‐fibrotic cell functions, such as *Hoxa11*, *Sall1*, sprouty receptor tyrosine kinase signaling antagonist 1 (*Spry1*), and prostaglandin reductase 1 (*Ptgr1*) (Figure 1j, green Venn diagram). Most prominent among the 38 DEGs persistently upregulated in stiff‐primed MSCs (5w100 ≥ 3w100) were genes with pro‐fibrotic and ECM‐related functions, including gremlin 1 (*Grem1*), thrombospondin 2 (*Thbs2*), and osteopontin (secreted phosphoprotein 1, *Spp1*) (Figure 1j, red Venn diagram).

Unbiased gene ontology (GO) enrichment analysis of DEGs confirmed the association of genes enriched in soft‐primed MSCs with regeneration, and developmental signatures in 5‐week stiff‐primed MSCs (Figure S1a–f). Highly ranked GO terms identified for soft‐primed MSCs included “morphogenesis regulators,” “tissue development and regeneration”, “integrin, collagen, and ECM binding”, and “TF complexes” (Figure S1a,c,e). Conversely, GO terms for stiff‐primed MSCs involved processes related to fibrogenesis and osteogenesis, such as “fibroblast growth”, “osteogenesis”, “ECM organization”, and contractile components like “myosin complex,” and “bone morphogenetic protein signaling” (Figure S1b,d,e). Collectively, these data indicate that mechanical priming of MSCs induces global chromatin remodeling, with a higher frequency of accessible gene promoters under stiff priming. Priming for at least 3 weeks shifts the accessible gene loci toward active regulatory regions, correlating with increased gene transcription over more extended priming periods. Stiff priming progressively increases the abundance of DAL and DEGs associated with fibrosis and osteogenesis, whereas soft priming suppresses them. Conversely, soft priming enhances transcriptional patterns associated with regeneration and anti‐fibrotic functions.

### 2.2 Mechanically Induced Accessible Regions, Transcription, and Enriched Motifs Are Memorized

**FIGURE 2:**
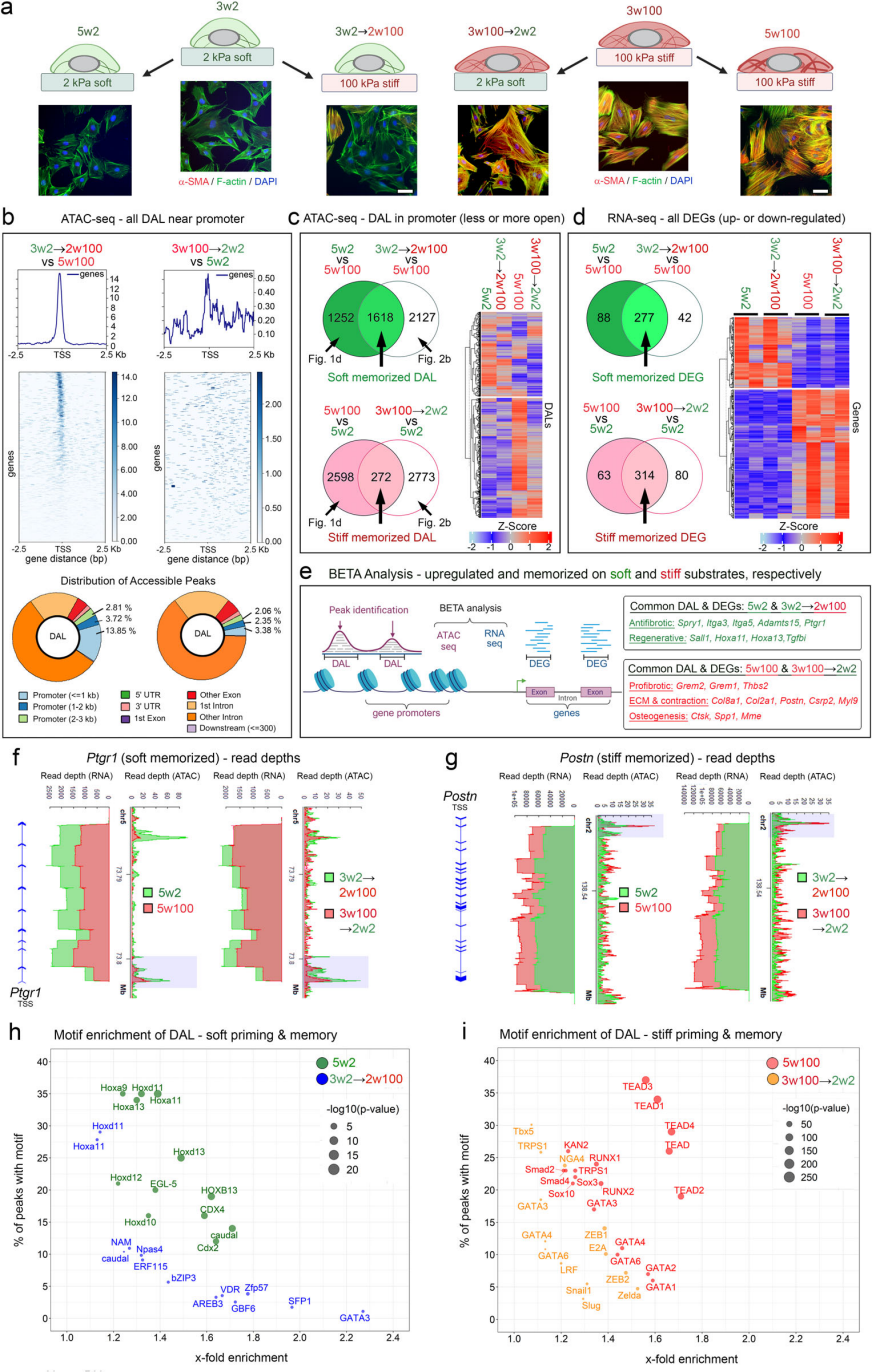
Soft and Stiff MSC Priming Manifests as Memorized Changes in Gene Accessibility and Expression (a) MSCs were mechanically primed for 3 weeks to establish memory. Mechanical memory was then assessed by switching 3‐week soft‐primed MSCs to a stiff environment for 2 weeks (3w2→2w100) and 3‐week stiff‐primed MSCs to a soft environment for 2 weeks (3w100→2w2). Persistent MF activation is indicated by the presence of α‐SMA (red) in stress fibers. Scale bar: 20 µm. (b) Heatmaps in blue scale show ATAC signal density within ±2.5 kb of gene transcription start sites (TSS) near (±2.5 kb) the promoter regions of genes. Shown are only genes with differentially accessible loci (DAL) (*p* ≤ 1e‐4), with each row representing one single gene. Compared are the ATAC‐seq data for MSCs with mechanical memory: 3w2→2w100 vs 5w100 for soft memory and 3w100→2w2 vs 5w2 for stiff memory. (c, d) Venn diagrams show the number of DAL within gene promoters (*p* ≤ 1e‐3 and ±5 kb of TSS) (c) and all differentially expressed genes (DEGs) (*p* < 0.05, log2‐fold change of >0) (d), shared between 5w2/5w100 and 3w2→2w100/5w100 for memory in soft environment (green), and 5w100/5w2 and 3w100→2w2/5w2 for memory in stiff environment (pink). Heatmaps (red‐blue scale) of DAL show the accessibility states of preserved regions (c), and DEGs show the expression patterns of memorized transcripts (d). Red hot color indicates open regions or highly expressed genes, and blue cold color represents closed regions or lowly expressed genes in mechanically primed MSCs. The heatmap of ATAC‐seq data shows the average normalized counts across two biological replicates. The RNA‐seq heatmap shows normalized counts for the two biological replicates. (e) Illustration of BETA application to connect DAL to DEG. Significant DAL‐DEG connections were scored on a rank product of DAL peak score (ATAC‐seq) and the adjusted p‐value (RNA‐seq) of any genes with a known TSS±2.5 kb from the DAL peak center. Examples of significant DAL‐DEG connections identified using *BETA* that were retained between 5w2 and 3w2→2w100 (green) or 5w100 and 3w100→2w2 (red) are highlighted in boxes. (f, g) Sushi plots show RNA‐seq and ATAC‐seq sequencing tracks for two selected genes: anti‐fibrotic *Ptgr1*, which shows higher expression in a soft environment, and the ECM gene Postn, which exhibits higher expression in a stiff environment (g). The ATAC‐seq and RNA‐seq data were normalized for sequencing depth, and the Y‐axis scale was adjusted to optimize peak visualization for each sample. Green tracks represent 5w2 and 3w2→2w100, while red tracks represent 5w100 and 3w100→2w2. The blue arrows show the direction of genes on the positive strand (downward) and the negative strand (upward), and the purple box indicates the differential accessibility around the *Ptgr1* and *Postn* promoters. (h, i) Motif enrichment analysis identifies candidate transcription factors specific to stiffness (5w2, 5w100) and mechanical memory (3w2→2w100, 3w100→2w2) for soft (h) and stiff environments (i). The percentage of peaks with motifs and the fold enrichment are plotted, and the circle size indicates significance levels. All ATAC‐seq data are shown for two biological replicates (MSCs from two different rats), and statistics for bulk RNA seq 5‐week priming and memory were calculated from 2 biological replicates (N = 2).

We then examined how changes in chromatin accessibility become lasting through mechanical priming and how they help preserve MSC gene transcription profiles after switching mechanical conditions. To assess mechanical memory, MSCs were primed for 3 weeks on either 2 kPa soft (3w2) or 100 kPa stiff surfaces (3w100), and then switched (→) to the other surface for another 2 weeks (Figure 2a, 3w2→2w100, 3w100→2w2). Consistent with our previous studies using the same setup [12, 13], 3‐week stiff priming was enough to imprint α‐SMA expression in stress fibers as a characteristic MF feature, which persisted even 2 weeks after switching to soft surfaces (Figure 2a, 3w100→2w2). Conversely, 3‐week soft priming not only suppressed MF activation during the priming period but also prevented MSC‐to‐MF mechanical activation after switching to stiff surfaces for two more weeks (Figure 2a, 3w2→2w100).

To identify memorized genes with highly accessible promoter regions in soft‐primed MSC, we followed this approach: (i) Consider only DAL within ±2.5 kb of the TSS, regardless of whether the loci were less or more accessible in the comparison. (ii) Find DAL that are common between soft growth (Figure 1d, 5w2 versus 5w100) and after transfer from soft to stiff surfaces (Figure 2b, 3w2→2w100 versus 5w100) (Figure 2c, green Venn diagrams). The overlap represents DAL in soft‐primed MSCs that kept their accessibility after the switch (‘soft memorized DAL’). (iii) Conduct the same analysis with the corresponding DEGs (Figure 2d). (iv) Match DAL with DEGs using *BETA* analysis [35] (Figures 1g, and 2e) and then focus on candidate genes that are more accessible, upregulated, and memorized in soft‐grown MSCs (Figure S2a–d, Figure 2c,d, heatmaps of upregulated genes, 5w2, 3w2→2w100, marked in red). The same analysis was used to identify stiff‐memorized genes (Figure 2c,d, red Venn diagrams).

Following this approach, we found: (i) a high amount and frequency of DAL around the TSS of genes compared between MSCs switched from soft to stiff (3w2→2w100) and always stiff‐grown MSCs (5w100) (Figure 2b, left heatmap and histogram); 20.03% of the DAL were detected within ±2.5 kb of the promoter (Figure 2b, left pie‐chart). Although these metrics were lower when comparing stiff‐memorized (3w100→2w2) and always soft‐grown MSCs (5w2), still 7.79% of the DAL were within the gene promoter (Figure 2b, right side). Thus, switching from soft to stiff results in a relatively higher DAL amount than the inverse surface switch. (ii) Of the 2,870 DAL in gene promoters differentially accessible between soft‐ and stiff‐grown MSCs (5w2 versus 5w100, Figure 1d), 1,618 DAL were common with the 3,745 DAL in gene promoters differentially accessible between stiff‐grown and switched from soft to stiff MSCs (3w2→2w100 versus 5w100, Figure 2b) (Figure 2c, green Venn diagram). We consider these 1,618 DAL ‘soft‐memorized’. In the Venn diagram created with the 3,045 DAL in gene promoters differentially accessible between switched from stiff to soft and soft‐grown MSCs (3w100→2w2 versus 5w2, Figure 2b), 272 DAL were ‘stiff‐memorized’ (Figure 2c, red Venn diagram). (iii) The same analysis performed with all DEGs delivered 277 soft‐memorized DEGs (Figure 2d, green Venn diagram) and 314 stiff‐memorized DEGs (Figure 2d, red Venn diagram). The blue‐red‐scaled heatmaps summarize the accessibility states of preserved regions (1,980 DAL in gene promoters, Figure 2c) and the expression patterns of the respective transcripts (591 memorized DEGs, Figure 2d).

(iv) Beta analysis linking DAL and DEG then delivered 14 genes that were persistently upregulated with open regions upon soft priming (Figure 2e, green). The list included genes that have been described as anti‐fibrotic, such as *Spry1*, α3 integrin (*Itga3*), a disintegrin and metalloprotease with thrombospondin type 1 motif 15 (*Adamts15*), *Ptgr1*, prostaglandin‐endoperoxide synthase 1 (*Ptgs1*), and regeneration‐associated genes like *Sall1*, *Hoxa11*, and *Hoxa13*. Other genes, like α5 integrin (*Itga5*) [36], and transforming growth factor beta induced (*Tgfbi* [37]) (Figure 2e, green), as well as collagen type XVII (*Col17a1*), rho GTPase activating protein 29 (*Arhgap29*), are related to tissue remodeling with context‐dependent pro‐ or anti‐fibrotic roles. Additionally identified ‘soft genes’ were scavenger receptor cysteine‐rich family member with 5 domains (*Scc5d*), synuclein gamma (*Sncg*) with yet unknown functions in tissue remodeling and fibrosis. In contrast, the 86 memorized genes found nearby persistently open regions after priming on stiff surfaces included mediators of fibrosis, ECM structure, contraction and scar formation (Figure 2e, red). Among the most prominent markers were *Grem2*, *Grem1*, and *Thbs2*, collagen type VIII α1 chain (*Col8a1*), collagen type II α1 chain (*Col2a1*), periostin (*Postn*), cysteine and glycine‐rich protein 2 (*Csrp2*), and myosin light chain 9 (*Myl9*), as well as genes characteristic of osteogenesis, such as cathepsin K (*Ctsk*), *Spp1*, and membrane metalloendopeptidase (*Mme*) (Figure 2e, red). *Ptgr1* (Figure 2f), *Postn* (Figure 2g), and other examples (Figure S2c–f) are shown in representative histograms of ATAC‐seq and RNA‐seq read depths. Myosin heavy chain 3 (*Myh3*) and Wnt family member 16 (*Wnt16*) are examples of mechanically regulated genes whose expression and accessibility are not memorized (Figure S2g,h).

In summary, a 3‐week soft priming period is sufficient to establish mechanical memory, maintaining anti‐fibrotic transcriptomic profiles and suppressing contractile MF and osteogenic profiles even after switching to stiff surfaces. Notably, a substantial number of gene promoter regions became more accessible during soft priming and remained accessible, i.e., memorized, after MSCs were transferred to stiff surfaces. These results suggest that epigenetic factors contribute to the maintenance of soft memory, in addition to previously identified factors related to stiff memory.

### 2.3 HOXA11 is a Mechanosensitive TF Driving Anti‐Fibrotic Gene Expression in a Soft Environment

Next, we performed Homer prediction analysis to identify TF binding motifs within the DAL enriched in soft‐grown (5w2) and soft‐primed MSCs switched to stiff surfaces (3w2→2w100) (Figure 2h). TFs that bind to motifs prominent in both conditions are expected to mediate soft memory. The DAL of soft‐grown MSCs were highly enriched for TFs from the HOX and CDX families (Figure 2h, green data points). However, only the binding motifs of HOXA11 and HOXD11 were predicted to be memorized after MSCs were switched to stiff surfaces (3w2→2w100) (Figure 2h, blue data points). Similarly, the DAL of stiff‐grown MSCs (5w100) were predicted to be highly accessible for the binding of TFs belonging to the transcriptional enhanced associate domain (TEAD) family, which are required for Yes‐associated protein (YAP) and transcriptional coactivator with PDZ‐binding motif (TAZ). YAP and TAZ promote the expression of mechano‐responsive MF genes [6, 16, 38, 39, 40] (Figure 2i, red data points). Binding motifs for TFs from the Runt‐related TF (RUNX) family were also enriched, aligning with the reported induction of MSC osteogenesis by mechanical stress [31, 41, 42]. However, among the TF binding motifs highly accessible in stiff‐grown MSCs (Figure 2i, red data points), only those predicted to bind GATA3, GATA4, and GATA6 maintained significantly increased accessibility after switching to soft surfaces (3w100→2w2). In other words, only the access for GATA family TFs was memorized (Figure 2i, orange data points).

**FIGURE 3:**
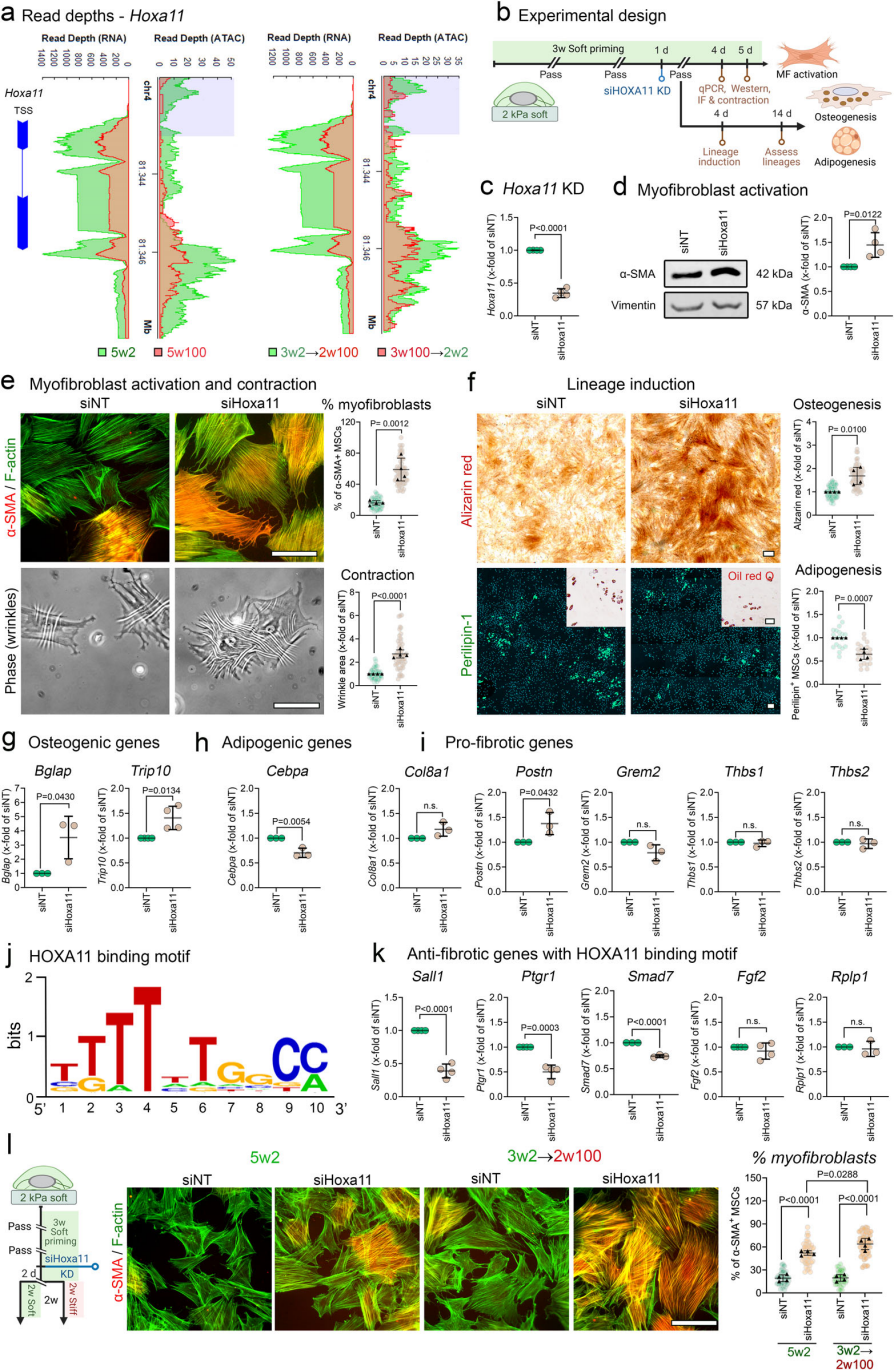
HOXA11 Is a Master Transcriptional Regulator of MSC Soft Memory (a) *Hoxa11* expression (RNA‐seq) and promoter accessibility (ATAC‐seq) were normalized for sequencing depth and displayed in Sushi plots. The blue arrow indicates gene transcription direction, with differential accessibility around the *Hoxa11* promoter shown within a purple box. Green tracks show data from soft‐grown (5w2) and soft‐to‐stiff switched (3w2→2w100) MSCs; red tracks show stiff‐grown (5w100) and stiff‐to‐soft switched (3w100→2w2) MSC data. (b) HOXA11 was knocked down (KD) in week 3 of MSC soft priming (3w2), using four siRNAs to assess acute MF activation 4–5 days after passaging, or lineage commitment after inducing adipogenesis or osteogenesis for 14 days. Non‐targeting siRNA (siNT) transfected MSCs were used as a control. After HOXA11 KD, MSC lysates were collected for (c) qRT‐PCR of *Hoxa11* expression and (d) Western blotting for α‐SMA, using vimentin as housekeeping. (e) After 5 days of HOXA11 KD, MSCs were stained for α‐SMA (red) and F‐actin (green) to determine the percentage of α‐SMA stress fiber‐positive MFs. MF contraction was quantified by seeding MSCs on pliable substrates that wrinkle under cell force; the wrinkle area percentage was measured from live phase contrast images after 24 h. (f) To assess lineage potential, HOXA11 KD MSCs were passed on tissue culture dishes and induced to undergo osteogenesis or adipogenesis. Osteogenesis was evaluated by Alizarin Red staining for calcium‐rich nodules, and adipogenesis was evaluated by immunostaining for perilipin‐1 or by Oil Red O staining for lipid droplets (insets). Soft‐primed MSCs were also analyzed via qRT‐PCR after 5 days of HOXA11 KD and siNT transfection, measuring genes for (g) osteogenesis, (h)adipogenesis, and (i) fibrogenesis. (j) Sequence logo representing the HOXA11 DNA binding motif, showing nucleotide preferences at each position. (k) qRT‐PCR analyzed anti‐fibrotic gene levels with HOXA11 binding motif after 5 days of HOXA11 KD. Rplp1 was a control, unaffected by mechanical cues and unlikely to have the HOXA11 motif. (l) After soft priming and 2 days before stiff surface switch in week 3 (3w2), MSCs underwent HOXA11 KD or siNT and remained on soft or switched to stiff surfaces for 2 more weeks. HOXA11 KD effects on soft mechanical memory were assessed after 5 days in week 5 by immunostaining for α‐SMA (red) and F‐actin (green), and α‐SMA stress fibers were quantified. Scale bars: 100 µm. Data are mean values (±standard deviation, SD) of at least three independent biological replicates (N = 3). Individual data points overlaid in the qRT‐PCR and Western blotting charts represent experimental replicates. qRT‐PCR data are first normalized to the housekeeping gene *Rplp3a*, and Western blotting data to vimentin; both are subsequently normalized to the siNT control. Circular data points in (e), (f), and (l) represent one image field, except for phase (wrinkles) in (e), where each dot represents one cell. Black triangles show the mean values calculated over all image fields per experimental repeat, with their SDs. Statistical significance was determined for the experimental mean values using Student's *t*‐test or repeated measures analysis of variance (ANOVA) with Šidák's post hoc test (n.s., not significant; *p* < 0.05 considered significant).

Of the two potential keepers of soft memory, HOXD11 and HOXA11, we focused on the latter because: (i) chromatin accessibility around the TSS of the *Hoxa11* promoter was higher in soft‐grown compared to stiff‐grown MSCs (Figures 3a and 1c,d), (ii) the chromatin accessibility status of *Hoxa11* was memorized after switching MSCs from soft to stiff surfaces (Figures 3a and 2c–e), (iii) *Hoxa11* gene transcription was consistently higher in soft‐grown MSCs and maintained after transfer to stiff surfaces (Figure 3a, Figure S3a, Figure 1f–j). (iv) The limited studies on HOXA11's role in MSCs suggest its expression relates to MSC subpopulations with improved healing potential in fractured bones, spleen, and endometrium [43, 44, 45]. To test if HOXA11 acutely regulates (suppresses) MSC‐to‐MF activation, we KD HOXA11 in soft‐primed MSCs (3w2) using a silencing RNA (siRNA) pool (Figure 3b). Reducing *Hoxa11* expression to 40% after 4 days KD (Figure 3c) resulted in a 1.4‐fold increase in α‐SMA expression in MSCs on soft surfaces (Figure 3d). Likewise, HOXA11 KD resulted in a 3.6‐fold higher percentage of α‐SMA stress fiber‐positive MSCs (Figure 3e), along with increased contraction, measured by quantifying phase‐bright deformations (wrinkles) on pliable substrates (Figure 3e). MSCs treated with non‐targeting siRNA (siNT) served as controls (Figure 3e).

As additional indicators of mechanically controlled MSC fate decisions, we evaluated adipogenic and osteogenic potential [31, 41, 42]. MSC osteogenesis on stiff surfaces and adipogenesis on soft surfaces are widely used as indicators of mechanically driven opposite lineage choices [41]. MSC fibrogenesis is linked to osteogenesis [31], and genes driving adipogenesis have been shown to antagonize fibrotic processes [46, 47]. After HOXA11 KD, soft‐primed MSCs were stimulated to undergo adipogenesis and osteogenesis following their respective fate‐induction protocols [48] (Figure 3b). HOXA11 KD led to a 1.7‐fold increase in osteogenesis, demonstrated by greater Alizarin Red staining of calcium‐rich deposits (Figure 3f). Conversely, MSC adipogenesis was reduced by 1.5‐fold, shown by decreased Oil Red O staining of lipid droplets and a lower percentage of perilipin‐1‐positive MSCs (Figure 3f). Even without chemically inducing lineage fate, KD of HOXA11 in soft‐primed MSCs alone resulted in increased expression of osteogenic markers, such as bone γ‐carboxyglutamate protein (*Bglap*) (3.5‐fold) and thyroid hormone receptor interactor 10 (*Trip10*) (1.4‐fold) (Figure 3g). Conversely, the adipogenic marker gene CCAAT enhancer binding protein‐α (*Cebpa*) was downregulated 1.4‐fold (Figure 3h).

Because mechanically regulated fibrogenic mediators like *Col8a1*, *Postn*, *Grem2*, and *Thbs1* (Figure 2b–e) were not significantly affected by HOXA11 KD (Figure 3i), we instead searched for HOXA11‐regulated (enhanced) anti‐fibrotic genes. The search was limited to genes consistently upregulated in soft‐primed MSCs (Figure 1e–j) with highly accessible DAL around the TSS of their promoters (Figure 1b–e) and a high frequency of predicted HOXA11 binding motifs (Figure 3j, Figure S3b). For this, we used MEME Suite's find individual motif occurrences (FIMO) algorithm with low p‐values indicating high prediction accuracy [49]; *Rplp1* served as a control gene that was not subject to mechanical regulation and does not contain the HOXA11 binding motif in its promoter (*p* ≤ 0.95). The top genes matching these criteria included known anti‐fibrotic mediators such as *Smad7*, *Fgf2*, and *Smurf2*, as well as genes not previously described as inhibitors of MF activation, like *Ptgr1* and *Sall1* (Figure S3). Consistent with most motif predictions, KD of HOXA11 resulted in reduced expression of *Smad7* (1.35‐fold), *Ptgr1* (2‐fold), and *Sall1* (2.6‐fold) in soft‐primed MSCs (Figure 3l). Control *Rplp1* (no change), *Fgf2* (1.1‐fold), and *Smurf2* (no change) did not show significant changes upon HOXA11 KD (Figure 3l). Therefore, lowering the elevated HOXA11 levels induced by soft priming enables MSCs to develop pro‐fibrotic and osteogenic traits even in a soft environment, likely through the suppression of anti‐fibrotic genes.

Having shown that KD of HOXA11 acutely induces MSC‐to‐MF activation on soft surfaces, we evaluated whether HOXA11 KD exposure to stiff surfaces exacerbates the effect of HOXA11 KD, as an indication of mechanical memory loss (Figure 3l). Transient HOXA11 KD in week 3 of soft priming was enough to sustain the acutely induced MF activation levels for an additional 2 weeks on soft surfaces (Figure 3l, 5w2). Notably, switching to stiff surfaces further enhanced the MF‐inducing effect of HOXA11 KD (1.25‐fold) (Figure 3l, 3w2→2w100). Overall, our data identify HOXA11 as a new TF that persistently inhibits MF activation by promoting the expression of anti‐fibrotic genes. Although HOXA11 acts as a suppressor of osteogenic markers, it does not directly inhibit the transcription of mechano‐responsive pro‐fibrotic genes. Therefore, we next examined whether previously unexplored HOXA11 target genes could help suppress pro‐fibrotic programs in MSCs.

### 2.4 Suppression of MSC‐to‐MF Activation by HOXA11 Is Mediated and Memorized Through SALL1

**FIGURE 4:**
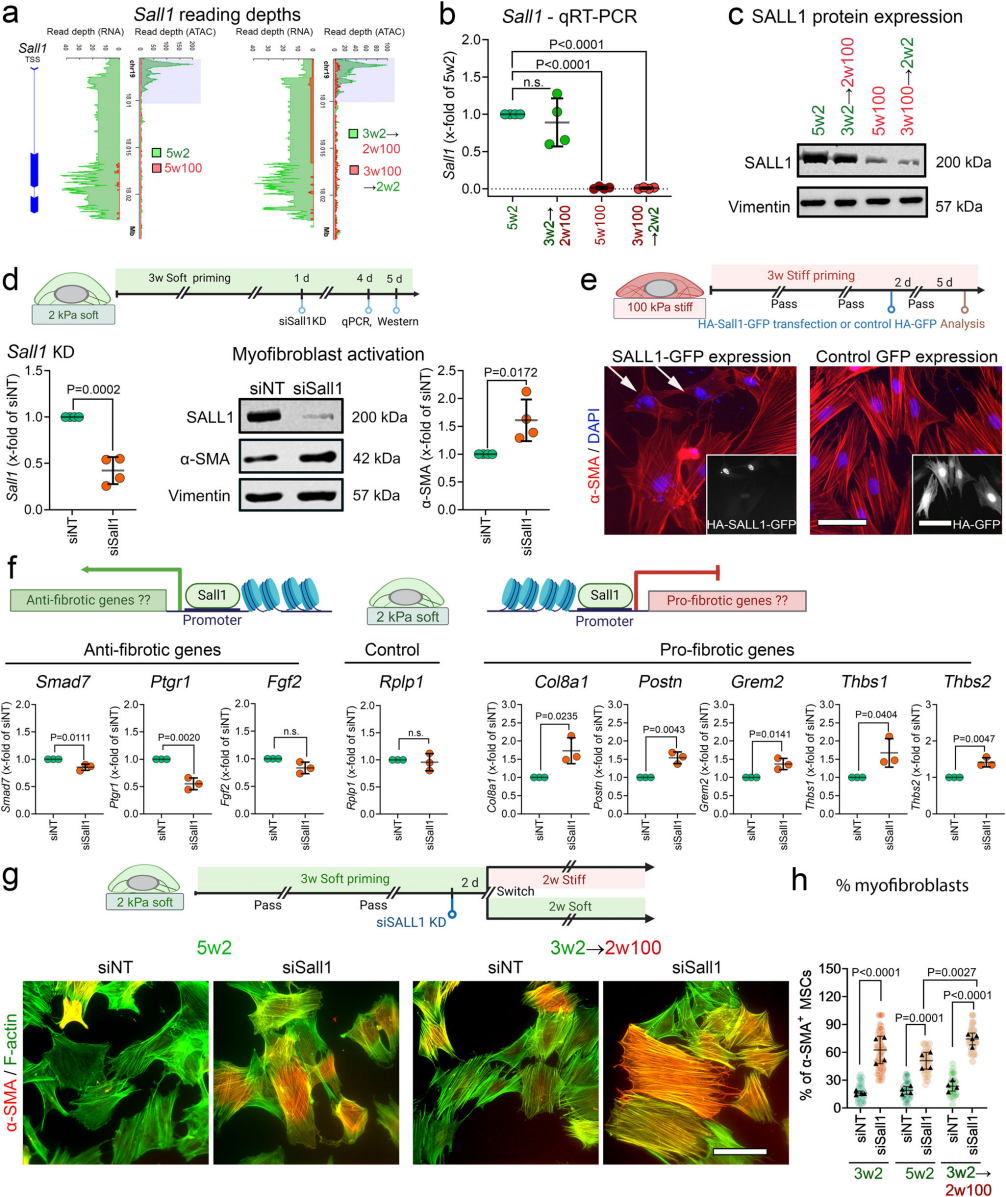
SALL1 Prevents the Mechanical Activation of MSCs (a) *Sall1* expression (RNA‐seq) and promoter accessibility (ATAC‐seq) were normalized for sequencing depth and displayed in Sushi plots. The gene transcription direction is shown with a blue arrow, and the differential accessibility around the *Sall1* promoter is shown in a purple box. Green tracks show data from soft‐grown (5w2) and soft‐to‐stiff switched (3w2→2w100) MSCs; red tracks show stiff‐grown (5w100) and stiff‐to‐soft switched (3w100→2w2) MSCs data. Lysates of MSCs were collected to (b) validate SALL1 expression using qRT‐PCR and (c) Western blotting with vimentin as a housekeeping protein. (d) SALL1 was knocked down (KD) in week 3 of MSC soft priming using a pool of four siRNAs to assess acute MF activation 4–5 days after passaging. Non‐targeting siRNA (siNT)‐transfected MSCs were used as a control. After SALL1 KD, MSC lysates were collected for qRT‐PCR analysis of *Sall1* expression and Western blotting for α‐SMA and SALL1, with vimentin as a housekeeping control. (e) Stiff‐primed MSCs were transfected with either a human SALL1 construct tagged with green fluorescent protein (GFP) and hemagglutinin (HA), or with a control GFP‐HA construct alone. Human SALL1‐GFP‐HA and control GFP‐HA OE were performed at week 3 (3w100), followed by passaging after 2 days and sample collection for immunofluorescence staining after 5 days. The transfected MSCs were stained for HA (green), α‐SMA (red), and DAPI to assess α‐SMA stress fiber‐positive MFs. (f) qRT‐PCR further assessed soft‐primed MSCs after 5 days of SALL1 KD and siNT‐transfection for the expression of anti‐fibrotic, control, and pro‐fibrotic genes. (g) After soft priming and 2 days before stiff surface switching in week 3 (3w2), MSCs were transfected with siSall1 or siNT and kept for two additional weeks to assess the effects of SALL1 KD on MF activity for soft mechanical memory. (h) After 5 days in week 5, MSCs were stained for α‐SMA (red) and F‐actin (green), followed by quantification of α‐SMA stress fiber‐positive MFs. To account for differences in the expression levels and molecular weights of SALL1 and vimentin, equal relative protein amounts were loaded onto separate gels, and each protein was detected individually. All scale bars: 100 µm. Independent experiments were performed with cells from at least 3 different rats. All graphs show mean values (±standard deviation, SD) of at least 3 independent biological replicates (N = 3). qRT‐PCR data are first normalized to the housekeeping gene *Rplp3a*, and Western blotting data to vimentin; both are subsequently normalized to the siNT control. Each circular data point in (h) represents one image field. Black triangles show the mean values calculated across all image fields per experimental repeat, with their SDs. Statistical significance was determined for the experimental mean values using Student's *t*‐test or repeated measures analysis of variance (ANOVA) with Šidák's post hoc test, except for (b), which was analyzed with Dunnett's test (n.s., not significant; *p* < 0.05 considered significant).

Of the two genes with predicted HOXA11 promoter binding motifs that were most strongly down‐regulated by HOXA11 KD in soft‐primed MSCs (Figure 3k, *Ptgr1*, *Sall1*), we focused on *Sall1* because: (i) HOXA11 binding motifs in the accessible *Sall1* promoter were significantly enriched (Figure S3b); (ii) *Sall1* was the most significantly and persistently up‐regulated gene in soft‐ versus stiff‐grown MSCs (Figure 1f,g, Figure S3a); (iii) the *Sall1* gene promoter became progressively more accessible over time with priming (Figure S3c); (iv) *Sall1* expression patterns were memorized after switching mechanical conditions (Figure 1i,j, Figure S3a); (v) joint RNA‐seq and ATAC‐seq analysis showed that highly transcribed and highly accessible regions in the *Sall1* promoter are consistent in soft‐grown MSCs, even after substrate switch (Figure 4a); and (vi) high *Sall1* expression in soft‐grown MSCs links various pathways in GO enrichment analysis, including regeneration, transcriptional complexes, and transcriptional repressor complexes (Figure S2a,c,e), supporting its proposed role in maintaining progenitor cell stemness [50].

High *Sall1* expression observed in bulk RNA‐seq of soft‐grown (5w2) (Figure 1) and soft‐primed switched MSCs (3w2→2w100) (Figure 2) was confirmed by qRT‐PCR (Figure 4b) and Western blotting (Figure 4c). In contrast, SALL1 was nearly absent in stiff‐grown MSCs (5w100) and not induced by switching from stiff to soft surfaces (3w100→2w2) (Figure 4c). Subsequently, we knocked down SALL1 in 3‐week soft‐primed MSCs to assess its role in suppressing transient MF activation (Figure 4d). SALL1 KD decreased *Sall1* mRNA and SALL1 protein levels to 42%, leading to a 1.5‐fold increase in α‐SMA expression in soft‐primed MSCs relative to siNT controls (Figure 4d). Conversely, overexpressing SALL1 in stiff‐primed MSCs (3w100) reduced α‐SMA expression in stress fibers compared to the control plasmid and non‐transfected MSCs (Figure 4e).

In principle, the TF SALL1 can suppress MF activation by either activating anti‐fibrotic genes or repressing pro‐fibrotic genes (Figure 4f). Among the anti‐fibrotic genes with predicted SALL1 binding motifs (Figure S4a–c) that were highly expressed and memorized by soft priming (Figure 2e), only *Ptgr1* was significantly reduced (1.8‐fold) by SALL1 KD (Figure 4f). In contrast, SALL1 KD greatly increased the expression of several pro‐fibrotic genes in soft‐primed MSCs, including *Col8a1* (1.7‐fold), *Postn* (1.5‐fold), *Grem2* (1.4‐fold), *Thbs1* (1.7‐fold), and *Thbs2* (1.4‐fold). Like done for HOXA11, we tested whether SALL1 KD would sensitize soft‐primed MSCs to MF activation upon exposure to stiff surfaces (Figure 4g). Indeed, SALL1 KD in week 3 of soft priming maintained acutely induced α‐SMA‐positive stress fibers for 2 more weeks on soft surfaces (Figure 4g,h, 5w2). However, unlike results obtained after HOXA11 KD, depleting MSCs of SALL1 during soft priming not only lifted the block on fibrogenesis but also enhanced MF induction (1.5‐fold) by switching to stiff surfaces (Figure 4g,h, 3w2→2w100). Overall, these results support SALL1 as a new mechano‐responsive and HOXA11‐controlled TF that persistently inhibits MF activation by acting as a suppressor of pro‐fibrotic gene expression on soft surfaces and is memorized after the transition to stiff surfaces.

### 2.5 GATA6 is a Keeper of Mechanical MF Memory in Stiff‐Primed MSCs

**FIGURE 5:**
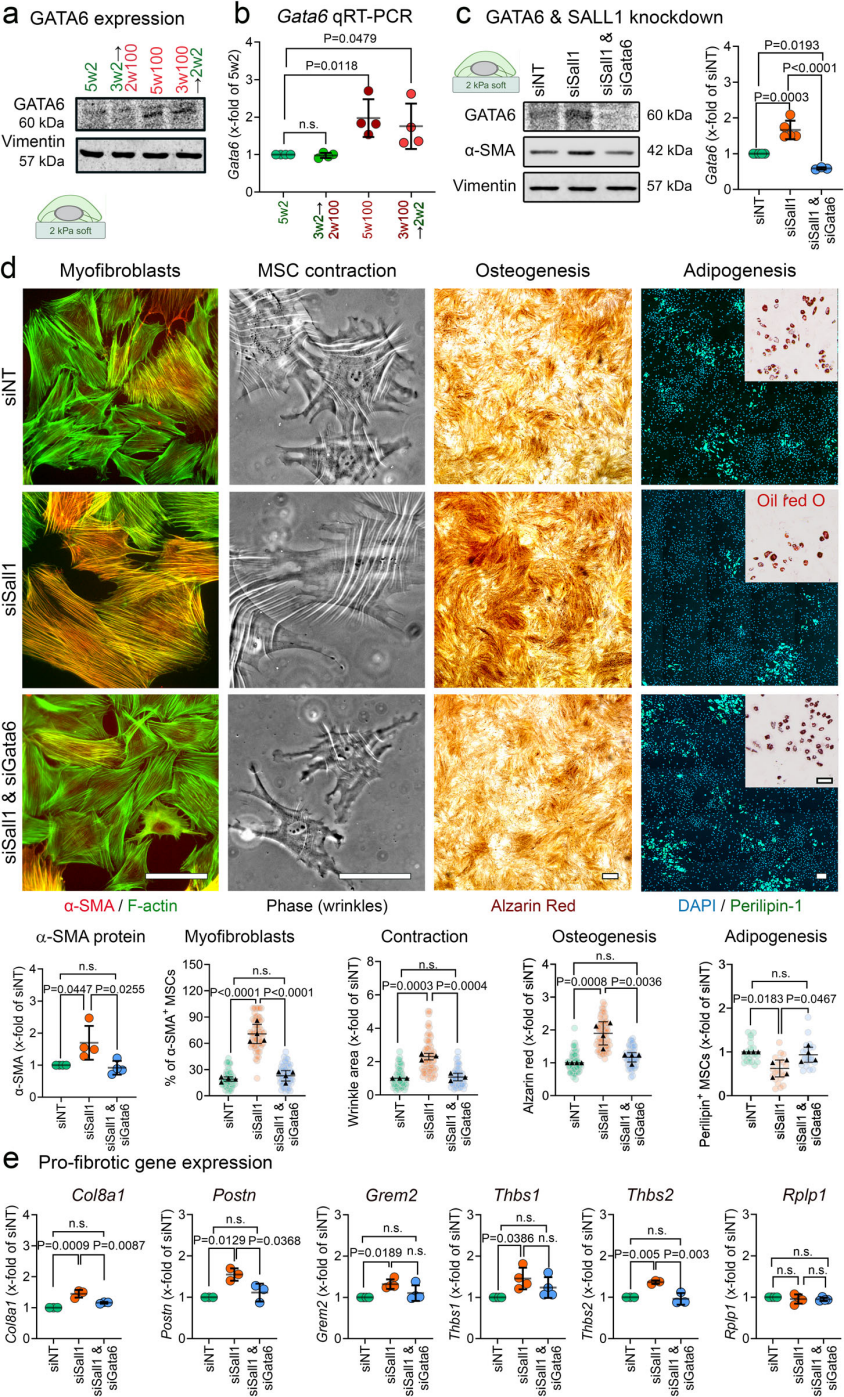
The Absence of SALL1 Permits GATA6‐driven MF Activation After collection of lysates of soft‐grown (5w2), soft‐to‐stiff switched (3w2→2w100), stiff‐grown (5w100), and stiff‐to‐soft switched (3w100→2w2) MSCs, GATA6 expression was assessed using (a) qRT‐PCR and (b) Western blotting with vimentin as housekeeping protein. (c‐e) Either SALL11 or joint SALL1 and GATA6 knockdown (KD) was performed in week 3 of MSC soft priming (3w2) using a pool of four siRNAs to assess acute MF activation 4–5 days after passaging, or lineage commitment after inducing adipogenesis or osteogenesis for 14 days. Non‐targeting siRNA (siNT)‐transfected MSCs were used as a control. After SALL1 or double SALL1 and GATA6 KD, MSC lysates were collected for (c) Western blotting for α‐smooth muscle actin (α‐SMA) and GATA6 expression, using vimentin as housekeeping protein, and (d) qRT‐PCR analysis of *Gata6* expression. Following 5 days KD, MSCs were stained for α‐SMA (red) and F‐actin (green) to calculate the percentage of α‐SMA stress fiber‐positive MFs. To quantify MF contraction, MSCs were seeded onto pliable surfaces that wrinkle under cell force, and the percentage of the wrinkle area (bright lines) attributable to MF activation was quantified from live phase contrast images after 24 h. To assess lineage potential, MSCs were either KD for SALL1 or KD for both SALL1 and GATA6, then plated in tissue culture dishes and chemically induced to undergo osteogenesis or adipogenesis. Osteogenesis was assessed by staining calcium‐rich nodules with Alizarin Red, and adipogenesis was evaluated by immunostaining for perilipin‐1 (green) or using Oil Red O to detect lipid droplets (insets). (e) Soft‐primed MSCs were further assessed by qRT‐PCR after 5 days of KD for the expression of pro‐fibrotic genes and *Rplp1* as a control. To account for their similar molecular weights, GATA6 and vimentin were resolved and detected on separate SDS‐PAGE gels. All scale bars: 100 µm. Independent experiments were performed with cells from at least 3 different rats. All graphs show mean values (±standard deviation, SD) of at least 3 independent biological replicates (N = 3). Data points in qRT‐PCR and Western blot charts represent independent experiments, normalized to *Rplp3a* and vimentin, respectively, and then to siNT. Circular data points in image quantification represent one image field, except for phase (wrinkles), where each dot shows one cell. Black triangles show the mean values calculated across all image fields per experimental repeat, with their SDs. Statistical significance was determined for the experimental mean values using repeated measures analysis of variance (ANOVA) with Šidák's post hoc test, except for (b), which was analyzed with Dunnett's test (n.s., not significant; *p* < 0.05 considered significant).

As a potential pro‐fibrotic effector suppressed by SALL1, we identified the TF GATA6. Multiple members of the GATA family of TFs were predicted to be significantly enriched in the promoters of genes that were highly accessible (GATA1, 2, 3, 4, 6) and memorized (GATA3, 4, 6) in stiff‐primed MSCs (Figure 2i). Among these, only GATA6 protein and RNA were highly expressed in stiff‐primed MSCs and remained elevated after switching to soft surfaces (Figure 5a,b). Additionally, multiple SALL1 TF binding motifs were predicted in the Gata6 promoter (Figure S4d). Consistent with the proposed role of SALL1 as a suppressor of GATA6, SALL1 KD in 3‐week soft‐primed MSCs resulted in a significant increase in GATA6 mRNA and protein levels (1.6‐fold) (Figure 5c, Figure S5a). The increase in GATA6 expression following SALL1 KD was prevented by combined KD of SALL1 and GATA6 (Figure 5c). The dual KD of GATA6 and SALL1 also abolished the MF‐inducing effect of SALL1 KD alone by reducing α‐SMA expression by 1.9‐fold, the percentage of α‐SMA‐positive MSCs by 3.1‐fold, and MSC contraction by 2.1‐fold compared to SALL1 KD alone (Figure 5d). Simultaneously, the expression of mechano‐responsive pro‐fibrotic genes (*Col8a1*, *Postn*, *Grem2*, *Thbs1*, *Thbs2*) was increased by SALL1 single KD (Figure 4f), but not when GATA6 was KD at the same time (Figure 5e). Likewise, dual KD reduced calcium deposition during induced osteogenesis by approximately 1.5‐fold while increasing the percentage of perilipin‐1‐positive cells by 1.6‐fold during induced adipogenesis compared to SALL1 KD alone (Figure 5d). Therefore, the inhibitory effect of SALL1 on pro‐fibrotic gene expression is, at least in part, mediated through the suppression of GATA6.

**FIGURE 6:**
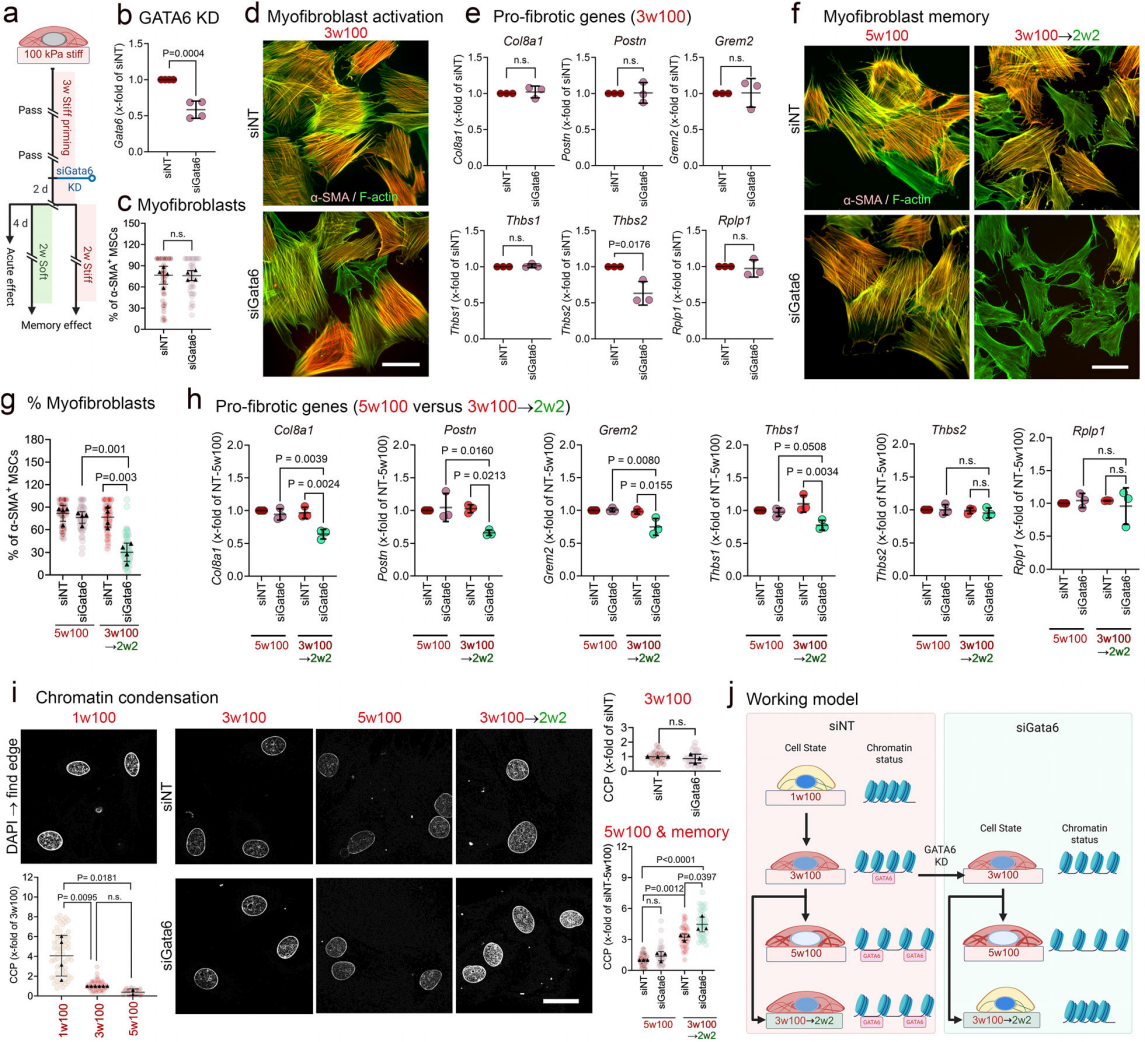
GATA6 Contributes to the Formation of MF Mechanical Memory (a) Acute and lasting effects of GATA6 knockdown (KD) were tested with stiff‐primed MSCs. To explore acute effects, MSCs were transfected with siGata6 or non‐targeting siRNA (siNT) after 5 days in week 3 of stiff priming. MSC lysates were collected for (b) qRT‐PCR analysis of *Gata6*  expression or (c, d) MSCs were stained for α‐smooth muscle actin (α‐SMA) (red) and F‐actin (green) to calculate the percentage of α‐SMA stress fiber‐positive MFs. (e) QRT‐PCR was used to assess stiff‐primed MSCs after 5 days of GATA6 KD for pro‐fibrotic gene expression. To assess the effects of GATA6 KD on mechanical memory, 3‐week stiff‐primed MSCs were transfected with siNT (control) and siGata6 (3w100), 2 days before switch to soft environment (3w100→2w2), or maintained on stiff surfaces (5w100) for an additional 2 weeks. (f, g) MSCs were assessed for  % of α‐SMA‐positive cells, and (h) expression of pro‐fibrotic genes. (i) Chromatin condensation was assessed using nuclear staining with DAPI and applying a find‐edge image processing algorithm to deliver a chromatin condensation parameter (CCP). CCP was evaluated in stiff‐primed MSCs over time of stiff priming and surface switch, following transfection of MSCs in week 3. Scale bars, d, f: 100 µm; i: 20 µm. (j) Working model of the potential mechanism of GATA6‐maintained stiff memory. Independent experiments were performed with cells from at least 3 different rats. All column graphs show mean values (±standard deviation, SD) of at least 3 independent biological replicates (N = 3). qRT‐PCR data are first normalized to the housekeeping gene *Rplp3a*, and then normalized to the siNT control. Circular data points in image quantifications represent a single image field, with statistics computed as the average across all image fields per experimental replicate. Black triangles show the mean values calculated across all image fields per experimental repeat, with their SDs. Statistical significance was determined for the experimental mean values using Student's *t*‐test or repeated measures analysis of variance (ANOVA) with Šidák's post hoc test (n.s., not significant; *p* < 0.05 considered significant).

Next, we evaluated whether KD of GATA6 in 3‐week stiff primed MSCs directly reduces MF activation (Figure 6a, ‘Acute effect’), as suggested by the enrichment of GATA6 binding motifs in pro‐fibrotic gene promoters (Figure S5b–d). Surprisingly, GATA6 KD by 50% (Figure 6b) did not alter the percentage of α‐SMA positive MSCs (Figure 6c,d) or the expression of pro‐fibrotic genes that were increased by SALL1 KD (Figures 6e and 4f). Motivated by our earlier findings that MF memory factors do not necessarily regulate acute MF activation [13], we then switched MSCs from stiff to soft surfaces (3w100→2w2) after GATA6 KD (Figure 6a, ‘Memory effect’). Indeed, GATA6 KD MSCs, but not siNT controls, showed a 2.4‐fold decrease in the percentage of α‐SMA‐positive MSCs (Figure 6f,g) and similarly reduced expression levels of pro‐fibrotic genes (Figure 6h) 2 weeks after switching to a soft surface. MSCs kept on stiff surfaces for the same duration (5w100) remained unaffected by GATA6 KD (Figure 6f–h). One exception was *Thbs2*, which was downregulated 1.6‐fold during acute GATA6 KD (Figure 6e) but not memorized 2 weeks after switching to soft surfaces or continued stiff culture (Figure 6h).

Next, we tested whether GATA6 overexpression, in turn, erased anti‐fibrotic soft memory in soft‐primed MSCs with low endogenous GATA6 expression (Figure S6a). Transfection of hemagglutinin (HA)‐tagged GATA6 (GATA6‐3×HA) into 3‐week soft‐primed MSCs enhanced GATA6 levels 12.4‐fold compared to MSCs transfected with a control GFP‐3×HA plasmid (Figure S6a). GATA6 overexpression alone did not induce MF activation in soft‐primed MSCs (3w2, 5w2) but resensitized them for MF activation upon switch to stiff surfaces (3w2→2w100), inducing a 2.7‐fold increase in MF activation compared to GFP–3×HA switched to stiff and GATA6–3×HA kept in a soft environment (Figure S6b). The question remained: how can GATA6 sustain MF memory without affecting acute MF activation? As a pioneer TF, GATA6 binding to gene promoters has been shown to enhance chromatin accessibility for other transcription regulators [51]. To explore this possibility and measure overall chromatin density changes, we calculated a chromatin condensation parameter (CCP) based on edge detection image enhancement of nuclear DAPI staining [15]. Consistent with published data [15], the CCPs of MSC nuclei decreased with increasing culture time on stiff surfaces by 11‐fold over 5 weeks (Figure 6i). GATA KD had no significant effect on chromatin density either acutely (3w100) or after 2 weeks of continued growth on stiff surfaces (5w100) (Figure 6i). In contrast, switching MSCs from stiff to soft surfaces (3w100→2w2) following GATA6 KD resulted in a 1.4‐fold increase in chromatin density compared to siNT controls (Figure 6i). Chromatin density also increased in soft‐switched control MSCs (Figure 6i, siNT, 3.2‐fold over 5w100 siNT), consistent with the reduction of accessible gene loci measured under these same conditions by ATAC‐seq (Figure 1b,c). As further evidence that GATA6 binding alone can increase gene accessibility by opening chromatin, we first measured the CCPs of 3‐week soft‐primed MSCs (Figure S7a, 3w2). Consistent with our ATAC‐seq data, baseline chromatin density was higher in soft‐ than in stiff‐primed MSCs (Figure S7b). SALL1 KD which enhanced GATA6 expression (Figure 5c) reduced the CCP of soft‐primed MSCs by 3.9‐fold, an effect prevented by combined KD of GATA6 (Figure S7c).

These findings led us to delineate how GATA6 regulates chromatin accessibility. GATA6 was previously shown to maintain chromatin accessibility by interacting with the histone acetyltransferases CREB‐binding protein (CBP) and p300. They acetylate histone H3 at lysine residue 27 (H3K27ac). H3K27ac is an active histone mark, associated with accessible promoter regions and chromatin openness [52, 53]. Our data support the connection between GATA6 and H3K27ac: 3‐week stiff‐primed MSCs exhibit a higher number of DAL (Figure 2c,d), less condensed chromatin (Figure S7a), higher GATA6 expression (Figure 5a), and 2.6‐fold higher H3K27ac levels (Figure S7d), compared with soft‐primed MSCs. To establish causal relationships, we KD GATA6, and subsequently analyzed H3K27ac levels after a surface switch (3w100→2w2). GATA6‐KD MSCs switched from stiff to soft surfaces, but not those maintained in stiff couture (5w100) showed a twofold reduction in H3K27ac levels (Figure S7e). These results suggest that GATA6 acts as a pioneer TF that contributes to pro‐fibrotic gene accessibility through H3K27ac‐mediated mechanisms. GATA6 KD without surface switch has no effect, likely because cytoskeletal stress on stiff surfaces is sufficient to unravel chromatin [33] and preserve H3K27ac levels. Additionally, GATA6 appears to directly regulate fibrosis‐related genes, such as *Thbs2*, independently of its memory role.

Motivated by these findings, we next explored whether the soft memory function of HOXA11 (Figure 3) can be explained by a similar pioneer TF function, as shown for other members of the HOX family [54]. Thus, we next measured chromatin accessibility and H3K27ac levels in 3‐week soft primed MSCs shortly (3 days) after HOXA11 KD, to minimize secondary effects on the expression of other pioneer TFs (Figure S7f). Acute KD of HOXA11 resulted in a 1.7‐fold increase in chromatin density, concomitant with a twofold decrease in H3K27ac (Figure S7g,h). These findings suggest that HOXA11 may modulate chromatin accessibility of anti‐fibrotic genes in soft‐primed MSCs by affecting H3K27ac marks in their promoters.

### 2.6 GATA6 and SALL1 Manipulation Alters MSC‐supported Skin Wound Healing

**FIGURE 7:**
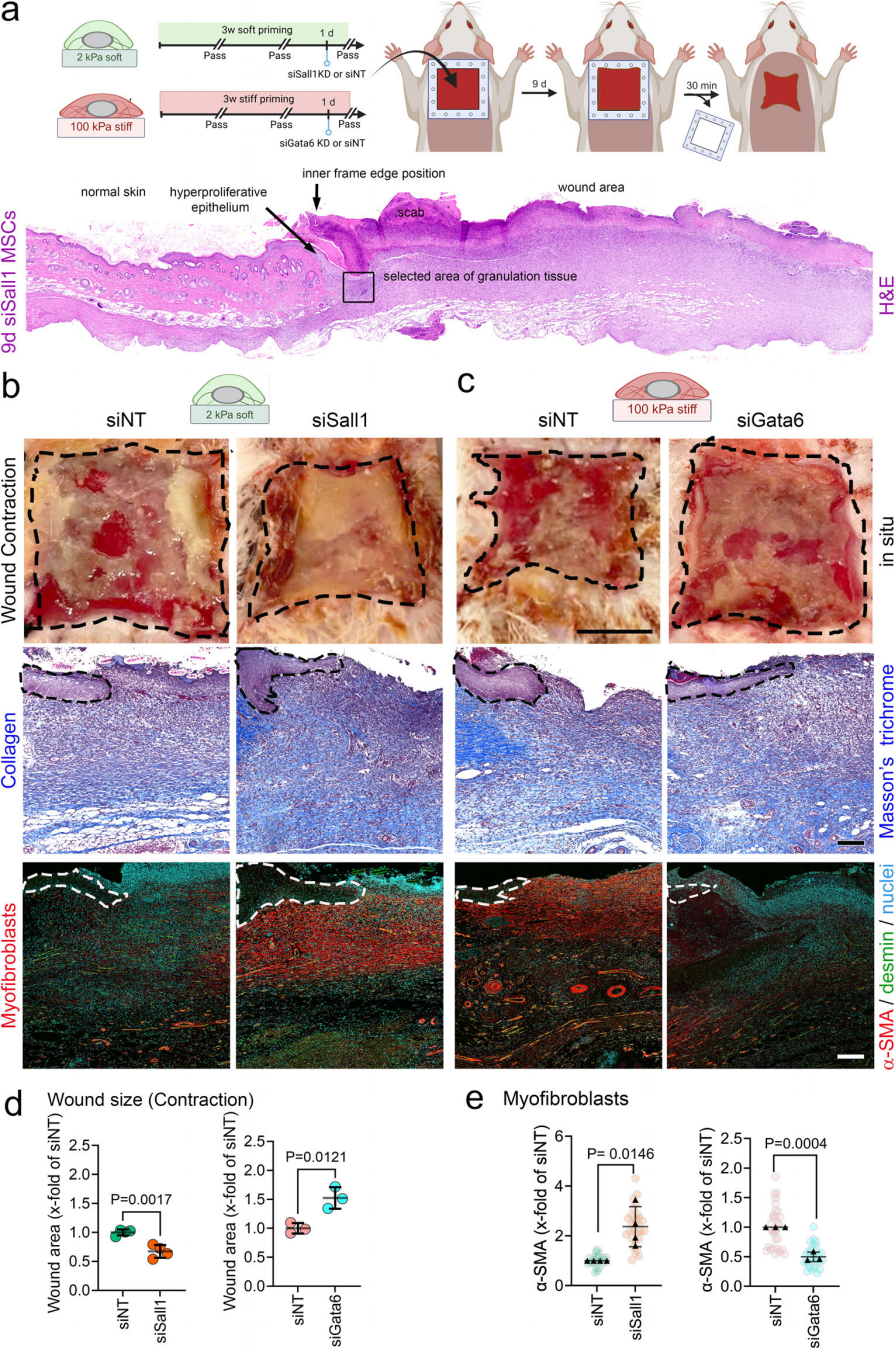
Manipulation of GATA6 and SALL1 Controls MSC‐supported Wound Repair (a) A rodent model of hypertrophic scarring was established by creating 25 × 25 mm full‐thickness excisional skin wounds on the backs of rats. Wounds were splinted by suturing the edges to a rigid plexiglass frame. 2 days before applying one million MSCs mixed with 1 ml of fibrin, sooft‐primed MSCs were transfected at week 3 with siSall1 to erase soft mechanical memory, or stiff‐primed MSCs were transfected with siGata6 to erase stiff mechanical memory. MSCs treated with non‐targeting siRNA (siNT) served as controls. H&E staining provides an overview of the wound. Wounds treated with (b) siSall1‐transfected soft‐primed MSCs or (c) siGata6‐transfected stiff‐primed MSCs were assessed 9 days post‐wounding for wound size, collagen amount by Masson's trichrome staining, and MF abundance by immunostaining for α‐smooth muscle actin (α‐SMA). (d) Wound contraction was quantified by measuring the percentage reduction in wound area 30 min after splint removal. (e) MF were quantified by measuring α‐SMA intensity at the wound edge. Scale bar, macroscopic wound: 1 cm, all others: 1 mm. Bar graphs represent mean values (± standard deviation, SD) of at least 3 independent biological replicates (N = 3). Circular Data points in image quantification (e) represent individual image fields. Black triangles show the mean values calculated across all image fields per experimental repeat, with their SDs. Statistical significance was determined for the experimental mean values using Student's *t*‐test (n.s., not significant; *p* < 0.05 considered significant).

Previously, we showed that transplanting soft‐primed MSCs reduces fibrotic traits in a rat model of skin hypertrophic scarring compared to wounds receiving stiff‐grown MSCs [13]. Here, we used the same model to test whether the mechanical memory of cultured MSCs can be overcome by manipulating anti‐fibrotic and pro‐fibrotic memory through SALL1 and GATA6, respectively. MSCs were soft‐ or stiff‐primed for 3 weeks before being transplanted onto dorsal rat wounds, which were splinted with a plastic frame that promotes fibrotic healing [55]. To erase memory, stiff‐primed MSCs were KD for SALL1 and soft‐primed MSCs were KD for GATA6 (and respective siNT controls) 2 days before mixing the cells into fibrin ECM for wound delivery (Figure 7a). Nine days post‐wounding and MSC transplantation, we measured MF contraction‐mediated wound size reduction 30 min after splint release, collagen accumulation using Masson's trichrome staining, and MF presence by staining α‐SMA in wound cross‐sections (Figure 7b). Wounds receiving SALL1‐depleted MSCs showed greater contraction, higher collagen content, and 2.5‐fold increase in α‐SMA‐positive MFs compared to wounds receiving soft‐primed control siNT MSCs (Figure 7b). These fibrotic features were similarly higher in wounds receiving control stiff‐primed MSCs. Still, they were reduced by delivering GATA6‐deleted stiff‐primed MSCs, which resembled wounds treated with soft‐primed MSCs (Figure 7c,d). Therefore, targeting SALL1 and GATA6 during MSC culture expansion represents a promising strategy to manipulate tissue repair and achieve desired wound‐healing outcomes.

## 3 Discussion

**FIGURE 8:**
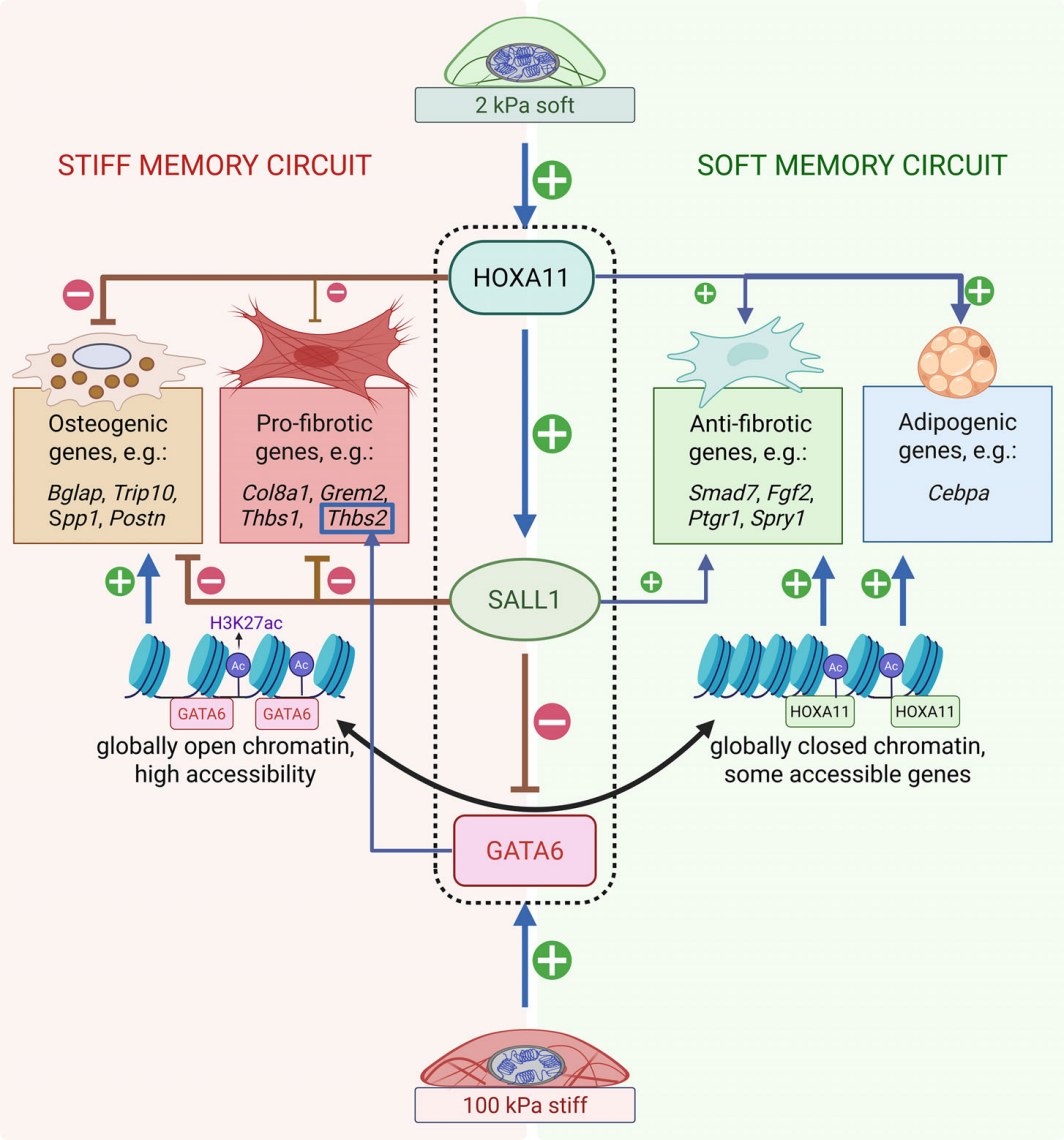
HOXA11‐SALL‐GATA6 Transcriptional Control Maintains MSC Mechanical Memory. The transcription profiles and chromatin accessibility landscape of MSCs undergo remodeling in response to physical cues. These cues induce a gene regulatory hub that includes transcription factors such as HOXA11, SALL1, and GATA6, as well as pro‐fibrotic and anti‐fibrotic genes. This hub consolidates regenerative versus myofibroblast phenotypes through mechanically controlled activation and interplay of these factors. In soft‐primed MSCs, *Hoxa11* modulates *Sall1* expression, which in turn dampens *Gata6* expression. Conversely, in stiff‐primed MSCs, downregulation of SALL1 expression increases GATA6 activity, driving profibrotic gene expression.

The memory of mesenchymal cells can be maintained through epigenetic modifications, which are heritable but reversible changes that alter gene expression without altering the DNA sequence [56, 57, 58]. Epigenetic modifications underlie the transition from transient MFs to persistent fibrotic MFs that can turn healthy repair into pathological scarring [12, 59, 60, 61, 62, 63, 64]. Consequently, epigenetic modifications are considered targets for reducing organ fibrosis [65, 66, 67, 68], including skin scarring [59, 69, 70, 71, 72]. Previously, we identified microRNA (miR) miR‐21 as a key factor in maintaining mechanically induced MF memory in MSCs, referred to as stiff memory. Now, we establish the HOXA11‐SALL1‐GATA6 axis as part of a transcriptional circuit that regulates the persistent suppression of pro‐fibrotic genes in a soft environment (Figure 8). We define such soft memory factors to be directly involved in persistently suppressing pro‐fibrotic and driving anti‐fibrotic features of MSCs. We proposed that soft (and stiff) memory factors are embedded in a complex network of hierarchical epigenetic mechanisms, including pioneer TFs, miRs, and DNA and histone modifications [9]. Our data reveal a mechanically controlled regulatory network in which HOXA11 transcriptionally regulates *Sall1*, leading to its accumulation in soft‐primed MSCs. SALL1 functions as a GATA6 suppressor and enables the transcription of pro‐fibrotic genes. Additionally, GATA6 and HOXA11 can act as pioneer TFs, respectively maintaining pro‐fibrotic and anti‐fibrotic gene accessibility for transcription, even after MSC stress conditions change. By manipulating GATA6 and SALL1 during cell expansion in culture, we engineered therapeutic MSCs with enhanced healing outcomes in hypertrophic rat wounds.

Extracellular mechanical stress directly influences the state of chromatin by the transmission of mechanical forces from the ECM to the nucleus through integrins and the contractile actin cytoskeleton [4, 9, 18, 23]. The prevailing observation is that high stress promotes overall chromatin decondensation by stretching DNA [15, 33, 73, 74]. In the context of MF activation, stress‐induced chromatin remodeling has been shown to increase the accessibility of gene regions containing binding motifs for TFs that drive MF activation and osteogenesis on stiff culture surfaces, such as YAP/TAZ and RUNX2 [24, 25, 74, 75]. Because extracellular and intracellular stresses balance each other [76], acute disruption or induction of cytoskeletal structure and tension decreases or increases gene accessibility peaks in ATAC‐seq, respectively [33]. Our data align with these findings, showing that the DNA of stiff‐primed MSCs exhibits more accessible peaks and higher signal magnitudes compared to soft‐primed MSCs. Notably, a significant portion (66%) of specific gene regions that become differentially accessible in stiff‐grown versus soft‐grown MSCs maintain their accessibility status even after changing mechanical conditions, indicating that the mechanically induced chromatin state is memorized. At least 8.6% of these mechanically controlled and memorized accessible DNA regions are near gene regulatory elements, positioning them as gene transcription regulators.

Contrary to the common observation that high stress leads to open chromatin, we discovered a small percentage of gene regions (1% of all DAL) that became more accessible after soft‐priming and maintained this chromatin state following a switch to stiff surfaces. These gene loci were thus soft‐memorized. Matching these open soft‐memorized regions to their corresponding soft‐memorized genes, we identified 14 genes that were specifically upregulated and memorized after soft‐growth. Among these, the TFs HOXA11 and SALL1 stand out as mechanosensitive factors that preserve MSC regenerative functions (Figure 8). KD of both HOXA11 and SALL1 in soft‐primed MSCs, along with overexpressing SALL1 in stiff‐primed MSCs, demonstrated their role in suppressing mechanically induced fibrogenesis and osteogenesis. Consistent with our finding that HOXA11 is a mechanosensitive TF, HOXA11 is among a group of genes upregulated in MSCs grown on aligned versus random topographies, alongside decreased expression of osteogenesis‐related genes [77]. Consistently, HOXA11 KD in our soft‐grown MSCs resulted in a significantly increased osteogenic potential and gene expression, supporting studies reporting elevated expression of ECM‐related genes when HOXA11 is absent in kidney cells [78]. Besides suppressing fibro‐ and osteogenic genes, binding motifs for HOXA11 are highly enriched in the promoters of genes with anti‐fibrotic functions that are upregulated and memorized in soft‐primed MSCs, such as *Ptgr1* and *Smad7*; all of which decreased following HOXA11 KD.

Moreover, HOXA11 binding motifs are predicted with high accuracy in the promoter of SALL1 and we confirmed HOXA11 as a potent inducer of SALL1 expression (Figure 8). A direct transcriptional relationship between HOXA11 and SALL1 in maintaining stem cell features has been suggested by studies that linked both TFs to shaping renal stem cell fates during kidney development [50, 78, 79]. Like HOXA11, SALL1 is involved in suppressing MF activation; chromatin regions predicted to bind SALL1 are more accessible in quiescent compared to MF‐activated hepatic fibroblasts [80]. In addition to acting as a transcriptional suppressor of fibrogenesis, SALL1 also regulates actin polymerization [81]. A truncated form of SALL1 that cannot translocate to the nucleus is linked to reduced stress fiber formation [81]. This cytosolic retention of SALL1 may explain why its overexpression in stiff‐grown MSCs significantly reduces α‐SMA stress fiber formation but does not affect *Acta2* expression.

How are specific regulatory regions of anti‐fibrotic genes made increasingly and consistently more accessible in a soft environment, and how do SALL1 and/or HOXA11 directly contribute to this process? Similarly, cells on soft surfaces were recently reported to have enhanced ATAC‐seq peaks near *BMF* gene of the Bcl‐2 modifying factor, a key transcriptional effector of adhesion‐dependent apoptosis—anoikis [82]. Others have produced enhanced ATAC‐seq peaks in genes related to differentiation by disrupting the LINC complex in epidermal cells [83]. These sites were rendered accessible by nuclear relaxation despite overall unchanged chromatin accessibility [83]. The molecular mechanisms through which nuclear relaxation can enhance chromatin accessibility remain unclear, but were linked to specific histone acetylation marks [83]. Since HOXA11 binding motifs are highly enriched in the accessible DAL in soft‐induced and memorized genes, we propose that HOXA11 functions as a pioneer TF. Pioneer TFs are activators of gene expression that open chromatin through DNA binding and recruit other components of the transcriptional machinery [19, 84]. Several members of the HOX family have been documented to play pioneer TF roles [54], and HOXA11 can bind to sites occupied by the pioneer TF HOXA13 during development [85]. It is tempting to speculate that HOXA11 plays a similar role in MSCs with soft memory by increasing chromatin accessibility near upregulated genes within the overall condensed chromatin environment of stress‐free MSCs.

While HOXA11 would be the first example of a soft‐controlled pioneer TF, the mechanosensitive TFs YAP/TAZ and RUNX2 have already been implicated as pioneer TFs to maintain stiff memory and MF activation in different mesenchymal cells [86, 87, 88]. In addition to their established role as mechanosensitive TFs, these have been shown to act as stiff memory factors by maintaining the force‐induced accessibility of regulatory regions of pro‐fibrotic and osteogenic genes after cell relaxation [13, 14, 15, 16, 86, 87, 88, 89, 90]. Consistent with these studies, gene loci characterized by TEAD binding motifs for YAP and TAZ, and those predicted to bind RUNX2, are highly accessible in our stiff‐primed MSCs. Interestingly, however, these loci are not subjected to stiff memory—that is, they are not maintained after switching MSCs from soft to stiff surfaces—and are unlikely to maintain stiff memory in our study.

Instead, we identified GATA6 as another mechanoresponsive pioneer TF and central mediator of persistent MF activation in MSCs (Figure 8). Our findings show that the increased accessibility of GATA6 binding motifs in stiff‐primed MSCs remains even after they transition to soft surfaces, along with the continued expression of GATA6 target genes. Overexpression of GATA6 erases soft memory and promotes MF activation after switching soft‐primed MSCs to stiff surfaces. Conversely, depleting GATA6 eliminates the pro‐fibrotic memory of stiff‐primed MSCs, which lose MF features after switching to a soft environment. Except for *Thbs2*, KD of GATA6 in MSCs grown continuously on stiff surfaces did not affect other pro‐fibrotic genes with predicted and accessible GATA6 binding motifs, such as *Postn*, *Col8a1*, *Grem2*, and *Thbs1*. However, all these genes, except *Thbs2*, still required GATA6 for maintenance following the transition to soft surfaces. Previous studies have already suggested that GATA6 is a pioneer TF: Its presence correlates with increased chromatin accessibility, while its absence limits access to other transcriptional regulators by closing gene loci in pluripotent stem cells [51, 52]. Similarly, GATA6 induction in our soft‐primed MSCs—indirectly through SALL1 KD—leads to widespread chromatin decondensation. In contrast, GATA6 depletion during stiff priming exacerbates the reduction in chromatin accessibility after MSCs are transferred to soft surfaces, compared with MSCs expressing GATA6. MSCs grown on stiff surfaces maintain globally open chromatin after GATA6 KD because persistent extracellular stress compensates for the loss of GATA6.

Overall, our results align with multiple reported roles of GATA6 in fibrosis progression, MF activation, and ECM production in heart and lung fibrosis [91, 92, 93, 94]. GATA6 is co‐expressed in the non‐proliferating, highly differentiated fibroblastic foci in human idiopathic pulmonary fibrosis [92], and inhibits fibroblast apoptosis, which contributes to sustained MF activity in tracheal fibrosis [91]. In MSCs, increased GATA6 expression during in vitro expansion has been associated with cell senescence and aging [95]. GATA6‐mediated induction of MF activation involves key fibrotic pathways such as Wnt/β‐catenin [91] and TGF‐β1 signaling [92]. GATA6 can also directly interact with SMAD2/3 [96]; future studies could explore how GATA6‐mediated chromatin remodeling increases the promoter accessibility of pro‐fibrotic genes for SMAD2/3 binding.

One unexpected finding of our study is that TEAD‐binding motif access for YAP and TAZ was not retained after switching stiff‐primed MSCs to soft surfaces, contrary to previous work [16]. A possible explanation is the different experimental approach and priming duration between studies that report YAP/TAZ as memory keepers and our study. First, during our week‐long priming, MSCs are lifted from the surfaces by trypsinization every week for passaging, i.e., MSCs are fully relaxed for ∼1 h in suspension before re‐adhering to a new surface. As an indicator of relaxation during trypsinization, the mechanosensitive MRTF‐A translocates from the nucleus to the cytosol upon MSC detachment and returns to the nucleus within a few hours at the start of each new passage on stiff surfaces [13]. For DNA memory to form, chromatin accessibility must be maintained during this relaxation phase. In contrast, other studies that assess mechanical memory by softening substrates via photomodulation do not detach the primed cells [97]. Second, the priming duration (dose) has been shown to significantly affect memory formation [16, 98, 99]. Simply put, the longer cells are mechanically primed, the stronger the memory imprinting, and the longer the memory persists. Consistent with this idea, only a few genes show expression changes after just 1 week of exposure to a stiff environment in our experiments. This duration aligns with the shorter priming periods of 3–10 days used in other studies [16, 74, 75]. While mechanical cues induce many accessible regions after 1 week of priming, most of these regions are not located near gene regulatory elements. However, after 3 and 5 weeks of priming, when MF activation peaks in stiff versus soft conditions [13], the number of DAL near promoters increases, leading to progressively altered transcription of the associated genes; these patterns persist in MSCs with mechanical memory.

While we focused on TF and pioneer TF control of mechanical memory, histone and DNA modifications are alternative and/or additional epigenetic mechanisms that regulate chromatin accessibility—and vice versa [100, 101, 102, 103, 104, 105, 106, 107]. For example, the lower chromatin condensation observed in MSCs on stiff compared to soft surfaces was shown to correlate with higher histone acetylation, increased expression of histone deacetylases, and reduced expression of acetyltransferases [15]. Cyclic tensile loading of MSCs causes high chromatin density mediated by the histone methyltransferase EZH2 [32]. Stiff environment also induces higher levels of histone H3 methylation at lysine residue K9 in human and lung fibroblasts [108]. All these DNA modifications persist after mechanical stress is removed and contribute to the mechanical memory that sustains persistent MF activation [15, 75, 109]. How GATA6 and HOXA11, as proposed pioneer TFs, cooperate with other epigenetic DNA modifications to establish and maintain mechanical MF memory remains to be shown. GATA6 has been reported to interact with histone acetyltransferases, including CBP/p300 [52]. Consistently, we measured reduced H3K27ac levels in GATA6 KD MSCs switched from stiff to soft surfaces. H3K27ac is an active histone mark associated with accessible chromatin regions [53], suggesting that GATA6 possibly maintains chromatin accessibility by preserving H3K27ac. Likewise, HOXA11 KD results in diminished chromatin accessibility and H3K27ac levels in soft‐grown MSCs. The molecular mechanisms directly linking GATA6 and HOXA11 to histone acetyltransferases remain to be explored.

Another open question is how high or low mechanical cell stress affects specific gene loci. For instance, why are the promoters of *Sall1* and *Hoxa11* more accessible on soft surfaces, and are *Gata6* promoter regions more accessible on stiff surfaces? One possibility is that regulatory chromatin regions of mechanosensitive genes are in stress‐exposed regions of the nucleus. For instance, cis‐regulatory regions located on DNA loops are not effective in regulating gene transcription when loop formation is controlled by stress [82]. Whether gene regulatory elements locate to nuclear regions exposed or protected from stress is further controlled by chromatin state, in turn regulated by histone acetylation and methylation and/or nuclear lamina mechanics [24, 73, 110, 111, 112]. Specificity can also be created at the level of stress perception, for instance by engaging different integrins. Our prior work has shown that MSCs and fibroblasts exhibit comparable stiffness‐dependent responses and priming when cultured on different ECM coatings, including fibronectin, pronectin, and gelatin [12, 13, 27]. However, a systematic study relating different ECM elastic properties, ECM ligands and their respective integrin receptors to specific epigenetic alterations is yet to be performed.

From a translational perspective, week‐long priming aligns well with typical MSC culture durations of 3–5 weeks, which are required to produce sufficient cells for a transplant [113]. Therefore, expanding MSCs on mechanically tuned culture surfaces is a practical strategy to generate MSCs with desired healing capabilities. In bioreactor systems that enable faster MSC expansion but make growth on soft surfaces more challenging, epigenetic regulation of MF memory offers an exciting alternative to create MSCs resistant to MF conversion when delivered to fibrotic environments, such as large area wounds. This resistance can be achieved, for example, by prior KD of GATA6, which reduced fibrotic markers in our rat transplant model. Conversely, KD of soft memory factors, such as SALL1, can yield MSCs that perform better under conditions of compromised healing, such as in chronic wounds. According to the Human Protein Atlas (https://www.humancellatlas.org/), SALL1, HOXA11, and GATA6 are also expressed in human MSCs and thus are amenable targets for human MSC therapies. HOX genes have been shown to guide cell fate restriction and location‐specific differentiation of fibroblasts during embryonic development, and this pattern is preserved into adulthood [114]. GATA6 has been implicated in MF activation, while SALL1 and HOXA11 have been associated with regenerative pathways, likely contributing to the preservation of progenitor function and stemness features within the native MSC niche [50, 78, 91].

Mechanical memory in MSCs is governed by both transcriptional networks and epigenomic mechanisms, not unlike MSC differentiation into distinct cell lineages [13, 15, 16, 32]. While epigenetic memory and differentiation rely on similar regulatory principles, mechanical memory can predispose MSCs toward specific lineage differentiation without inducing a fully differentiated state [13, 105, 115, 116, 117, 118]. As shown here and in our previous work, MSCs primed on stiff surfaces exhibit enhanced osteogenesis in osteogenic induction media. In contrast, MSCs primed on soft surfaces show increased adipogenesis following adipogenic induction. Yet, both mechanically induced states are still capable of making either lineage choice [13]. In other words, epigenetic mechanical memory is reversible, while differentiation is not.

Notably, controlling the stiffness of cell culture surfaces and mechanical memory influences not only contractile and ECM‐producing MF features but also a broad range of other transcriptional programs. We will continue to explore these as‐yet‐unknown mechano‐responsive gene patterns to elucidate how MSCs can be primed for wound healing. For example, transplanted MSCs are thought to act as ‘rheostats’ that produce ECM and cytokines to guide a normal wound‐healing process when the body's inherent systems have failed [2, 4]. It remains to be shown how mechanical memory affects MSC trophic actions and how these relate to traditional MF features, such as ECM production and scar contraction.

## 4 Materials and Methods

### 4.1 MSC Isolation and Subculture

The isolation of MSCs from rat bone marrow was approved by the Animal Care Committee of St. Michael's Research Vivarium (protocol #339). Female Wistar rats (200–230 g) (Charles River Breeding Laboratories) were euthanized by exposure to 5% isoflurane, followed by intracardiac injection of KCl. To isolate a single batch of MSCs, the femurs and tibias were collected from a single rat and cleaned of attached muscles. After cutting off both ends to expose the marrow cavities, the bones were centrifuged twice in culture medium at 10 x g for 10 min each, and the resulting cell pellets were resuspended. The bone marrow was then flushed with 10 mL of medium per bone using a syringe to detach any remaining MSCs. All suspended tissue and cell material was filtered through a 70 µm filter mesh and plated onto six 10 cm dishes with the desired pathophysiological stiffness. After an initial expansion passage on respective soft and stiff surfaces, MSCs were enriched by positive selection using anti‐CD54‐ and anti‐CD90‐conjugated magnetic beads to eliminate haematopoietic cells (Miltenyi Biotec), as before [13]. Cell proliferation was monitored by cell counting, comparing the initial seeding to cell numbers collected after one week. In passages 1–2 following the explant, MSC proliferation was moderately, but never significantly, higher on soft surfaces, whereas this trend was inverted in later passage MSCs. To offset these differences, cell seeding densities were adjusted between 1800 and 2000 cells cm^−2^. After one initial expansion passage on respective soft and stiff surfaces for 10 days, MSC purity was validated as described before [13]. MSC lineage‐induction potential was verified as previously published in detail [13] and shown in the results of this manuscript. MSCs were then subcultured at a seeding density of 2000 cells/cm^2^ and grown for 1 week between passages. At 80% confluence (after one week), MSCs were detached using trypsin‐EDTA (0.25%), resuspended in fresh medium, and seeded onto the respective surfaces using fresh silicone surface culture plates for passaging and/or experimental use. MSCs were isolated and maintained in α‐Minimum Essential Medium (α‐MEM) with L‐glutamine and nucleosides, supplemented with 10% fetal bovine serum and 1% penicillin and streptomycin (all culture products from Wisent Bioproducts).

For mechanical priming and memory formation, silicone elastomer substrates (Excellness Biotech SA) were used with elastic moduli of 2 kPa, mimicking soft healthy skin to prevent MF activation, and 100 kPa scar‐stiff to promote MSC‐to‐MF activation [13, 27]. Silicone substrates are non‐degradable and resistant to trypsin‐ or cell‐derived protease degradation. Silicones were routinely tested for endotoxin contamination using a limulus amebocyte lysate (LAL) test (Eagle Analytical Services). Results were consistently below the assay's detection limit (<1 endotoxin unit per surface). Before use, the substrate surfaces were sterilized, oxygenated, silanized, and coated with gelatin (2 µg/cm^2^; G9391, Sigma‐Aldrich) [119]. In our prior mechanobiology studies, we demonstrated similar priming results using collagen type I‐, fibronectin, pronectin‐, and gelatin‐coated silicone surfaces [12, 13, 27]. Gelatin was chosen here for cost‐effectiveness and its minimal effect on baseline MF activation [13]. MSCs were primed for 3 weeks (1 passage per week) on either soft or stiff surfaces [13]. To study mechanical memory, MSCs were initiated and primed on 2 kPa and 100 kPa surfaces, and were either soft‐primed for 3 weeks and then switched to stiff surfaces for another 2 weeks (3w2→2w100), or stiff‐primed for 3 weeks and then switched to soft surfaces for 2 more weeks (3w100→2w2). MSCs that were continuously grown for 5 weeks on stiff (5w100) and soft surfaces (5w2) served as memory controls.

### 4.2 RNA‐Seq, ATAC‐Seq, Promoter Binding Motif Analysis, and qRT‐PCR

To prepare RNA‐seq and ATAC‐seq libraries, RNA and DNA were collected concurrently at the end of the MSC culture periods defined above. ATAC‐seq libraries were generated from nuclei isolated from 100 000 MSCs following previously established protocols [120]. Samples were double‐barcoded using Nextera i7 and i5 indexes (Illumina), size selected to exclude fragments >600 bp using the SPRIselect system (Beckman‐Coulter), and pooled to a final concentration of 20 nM/library. Proper fragment size distribution was validated before sequencing via TapeStation (Agilent) as preliminary quality control to confirm that DNA fragment sizes followed the anticipated pattern of nucleosomal periodicity. For RNA‐seq, cells were lysed with TRIzol (Thermo Fisher) and RNA was isolated using Qiagen's RNeasy Micro Prep kit. Isolated RNA was converted into sequencing‐compatible libraries using the TruSeq Stranded mRNA Library Prep kit (Illumina). RNA isolates with masses ≥0.5 µg and RNA integrity number scores ≥9.0 were sequenced. Both RNA‐seq and pooled ATAC‐seq libraries were sequenced to a depth of ∼20 million paired‐end reads (2 × 150 bp) on the Illumina NovaSeq 6000 platform.

For ATAC‐seq data processing and analysis, the raw fastq files were first adapter‐trimmed and then subjected to preliminary quality control using *TrimGalore*! and *FastQC*, respectively [121, 122, 123]. Trimmed reads were mapped to the rn7 genome using the *Bowtie2* aligner [124]. Peaks were called on aligned reads using the –call‐peaks function from the *MACS2* package, and peak summits were identified as the region stratifying 100 bp upstream and downstream of each peak's center (201 bp total) [125]. For all samples, peak summits identified using *MACS2* were merged into a singular BAM file and converted into a count matrix of read accumulation within peak regions on a sample‐by‐sample basis using the package *featureCounts* [126]. Adapter trimming, quality control, alignment, peak calling, and count matrix quantification were all performed in a Linux environment. Count data were normalized with the R package *DESeq2* and used to generate MA plots [127]. The Linux package *Homer* was employed to quantify differential accessibility (*p* ≤ 1e‐4), and the resulting loci were annotated using the R package *GenomicRanges* [128, 129]. Heatmaps and distribution profiles of differentially accessible peaks within 2.5 kb of a known TSS were quantified and visualized using the Linux package *deepTools* [130]. The R package *Sushi* was applied to plot ATAC‐ and RNA‐sequencing traces along the rn7 genome [131]. Peaks with significant changes in accessibility were converted to FASTA format and evaluated for changes in TF binding site accessibility of known TF motifs using the FIMO tool within the *Homer* package. Peaks with non‐significant changes in accessibility for the same comparison were used as background.

For RNA‐seq data processing and analysis, Raw fastq files were first adapter‐trimmed and passed for preliminary quality control using the *TrimGalor*e! and *FastQC* packages, respectively [121, 122, 123, 132, 133].Trimmed reads were then mapped to the mRatBN7.2 genome using *HISAT2* and converted into gene count tables via FeatureCounts [126, 132, 133, 134]. All initial analysis was done by Galaxy EU platform, and then raw counts were used for downstream analyses using R. DEGs were detected using the package DESeq2 [127], and gene annotation was performed using the rn7 RefGene GTF file from Ensembl. Unsupervised learning via PCA was performed using variance‐stabilized *DESeq2* results [127]. Heatmaps of gene expression were generated using the *ComplexHeatmap* package from *DESeq2*‐normalized read counts after filtering out genes with a mean expression across all samples totaling fewer than 10 reads [135]. All enrichment analyses were performed using ShinyGO (version 0.81) and the geneXplain platform in September 2024, and Lollipop plot visualizations were performed using the dplyr and ggplot2 libraries. RNA‐seq and ATAC‐seq integration was performed using *BETA*. RNA‐seq differential expression results for all genes (*DESeq2*) and ATAC‐seq peak scores (*MACS2*) were converted to BETA‐compatible formats and submitted as inputs to the program. A window of 50 kb from the TSS was used to identify potential regulatory peaks, and all differentially expressed genes for a given condition were considered in the analysis. Significant peak‐expression associations were demarcated as genes that produced a Rank Product ≤1e‐3 [35]. To predict TF binding sites in gene promoters, we used the FIMO motif search tool in the MEME Suite [49]. Promoter regions with significant changes in accessibility were extracted from ATAC‐seq data. TF binding motifs for HOXA11 and GATA6 were obtained from Homer motif enrichment data, while the motif for SALL1 was retrieved from literature [136]. The predicted target genes were further validated using the Eukaryote Promoter Database, Harmonizome 3.0, and qRT‐PCR. Motif logos were generated using WebLogo to represent the consensus sequences of all predicted motifs with significant binding to promoter regions, as determined by FIMO analysis.

For qRT‐PCR, mRNA was extracted by the PureLink Mini RNA Kit (12183018A, Thermo Fisher Scientific) according to the manufacturer's instructions. RNA (1000–2000 ng) was reverse transcribed using SuperScript IV VILO Master Mix with ezDNase Enzyme (11766050, Invitrogen). PCR amplification was performed in triplicate by RT2 SYBR Green ROX (Qiagen, Redwood City, USA) by using StepOnePlus Real‐Time PCR System (Applied Biosystems) at 95°C for 10 min, 40 cycles at 95°C for 15 s, all at 60°C annealing temperature for 60 s, followed by the melt curve. Relative gene expressions were calculated by using rat *Rpl3a* as a reference gene, and the following rat‐specific primers were selected based on primer efficiency (90%–100%) and a single peak in the melt curve:

**Table:** 

*Bglap*	TCTGAGTCTGACAAAGCCTTCAT; GGGTCGAGTCCTGGAGAGTA
*Cebpa*	TGTACTGTATGTCGCCAGCC; GGTTTAGCATAGACGCGCAC
*Col8a1*	CAGCCAAGCCTAAATGTGATGG; CCTCGCAAACTGGCTAATGG
*Fgf2*	AGCGGCTCTACTGCAAGAAC; TGGAGCTGTAGTTTGACGTGT
*Gata6*	CTCACCGACCGTCAAGTCAA; AAGAATCCTGTCGCACCGAG
*Grem2*	TTGCCAAGAACGGGTTTCCT; GTCACTGTGTGGCAGACGTA
*Hoxa11*	CTCACCGACCGTCAAGTCAA; GAGAGTTTCAGGCCCCTTCC
*Postn*	GCTGCCATCACTTCTGACCT; GGTCCGTGAAAGTGGTTTGC
*Ptgr1*	CCTCTCCGTGGACCCTTACA; TTCTCCACCCTTCAAGCCAC
*Rplp1*	GCTTCTGTCTCTGAGCTTGCC; GAGCATTGATCTTATCCTCCGTGA
*Rplp3a*	CTGTGAGGGCATCAACATTTC; GTTGGTGTTCATCCGCTTTC
*Sall1*	ATTTCCAATCCGACCCCGAA; TGGACTCTTCCCTGTCGAGT
*Smad7*	ACCCCGATACAAAAACGGGAA; GCTGATCCAAAGGGGAAGG
*Thbs1*	TAACTGGGTTGTCCGCCATC; GGTGATTAGGAGTCTCGGCAC
*Thbs2*	TGTTCTGTCACCTGTGGCTC; GCCTCCATTCTGACGGATTCT
*Trip10*	GGGTACCGAGTTGTGGGATCA; TCGCATAAGACTGCTCCACC

### 4.3 KD and Overexpression Experiments

For all KD experiments, MSCs were transfected at 80% confluency on day 5 of a 3‐week soft or stiff priming regimen with 50 nM SMARTpool siRNAs (Horizon Discovery, Cambridge, UK). SiRNAs were directed against SALL1 (L‐093147), HOXA11 (L‐095969), GATA6 (L‐080135), or NT siRNAs (D‐001810). MSCs were subcultured at 2500 cells/cm^2^ 1 day after KD and analyzed 4–5 days later. To assess mechanical memory, KD of GATA6, SALL1, and HOXA11 was performed in week 3 of mechanical priming 2 days before switching to a new mechanical environment. All KD transfections were performed using Lipofectamine RNAi MAX (13778075, Invitrogen) with a 1:1 ratio of siRNA diluted in Opti‐MEM (31‐985‐070, Thermo Fisher Scientific) according to the manufacturer's protocol.

To overexpress human SALL1, MSCs were transfected at 80% confluency after 5 days in week 3 of stiff priming with HA‐SALL1‐GFP or control GFP‐HA piggyBac plasmid constructs. To overexpress rat GATA6, MSCs were transfected at 80% confluency with GATA6‐3×HA or control GFP‐3×HA piggyBac plasmid constructs after 3 days in week 3 of soft priming. Cells were co‐transfected with a PBase construct encoding piggyBac transposase for genomic integration. All overexpression transfections were performed using Lipofectamine Stem Transfection Reagent (STEM00015, Thermo Fisher Scientific) according to the manufacturer's protocol, with 1 µg of plasmid per transfection reaction.

### 4.4 Western Blotting

To detect α‐SMA, GATA6, and vimentin, MSCs were washed with PBS and scraped into a standard reducing lysis buffer containing 50 mM Tris (pH 6.8), 2% SDS, supplemented with Pierce Protease Inhibitor (Thermo Scientific A32955), and supplemented with 0.1% bromophenol blue, 10% glycerol, and 5% β‐mercaptoethanol after protein content quantification. To detect SALL1, we used a non‐reducing standard lysis buffer without β‐mercaptoethanol. Protein concentrations were measured using BCA Protein Assay Kits (Thermo Fisher Scientific), and equal amounts of protein were loaded after boiling the samples for 5 min at 95°C. Total proteins were separated by SDS‐polyacrylamide gel electrophoresis (8% gels for SALL1 detection, 15% gels for H3K27acand 10% gels for the other proteins) and wet transferred onto nitrocellulose or polyvinylidene fluoride membranes (for SALL1 and H3K27ac). The membranes were blocked in TBS buffer containing 5% milk, then incubated overnight at 4°C with primary antibodies diluted in the blocking solution. The primary antibodies used were: α‐SMA (1:500; SM1, mouse IgG2a; a kind gift from G. Gabbiani, University of Geneva, Switzerland), vimentin (1:2000; V9, mouse IgG1, Invitrogen), GATA6 (1:250; D61E4, rabbit IgG, Cell Signaling Technology), and SALL1 (1:250; rabbit polyclonal IgG, Thermo Fisher Scientific), H3K27ac (1:250; D5E4, rabbit monoclonal IgG, Cell Signaling Technology). After three 5 min washes with 0.1% Tween 20 in TBS (TBS‐T), the membranes were incubated for 1 h at room temperature with fluorescence‐conjugated secondary antibodies diluted 1:5000 in the blocking solution. The secondary antibodies used were anti‐rabbit IRDye 680RD and anti‐mouse IRDye 800CW (LI‐COR Inc.). To detect SALL1 and H3K27ac, HRP‐conjugated anti‐rabbit secondary antibodies (Invitrogen) were used, followed by chemiluminescent detection with SuperSignal Ultimate Sensitivity ECL Substrate (Thermo Fisher Scientific). Following three additional 5 min washes with TBS‐T, protein bands were visualized using a Li‐Cor Odyssey Fc system (Li‐Cor Biosciences). Bands corresponding to each protein were identified based on their molecular weight using a standard protein ladder (FroggaBio). Quantification of the blots was performed using ImageJ (US National Institutes of Health, Bethesda, MD, USA, http://imagej.nih.gov/ij/, 1997–2013) by measuring the integrated fluorescence intensity of each protein band. Band densities were normalized to the expression of housekeeping vimentin.

### 4.5 Osteogenic and Adipogenic Differentiation of MSCs

MSC lineage differentiation potential was measured after inducing osteogenesis and adipogenesis using established protocols [13, 137, 138]. For adipogenic differentiation, MSCs were seeded in plastic 12‐well plates at a density of 5000 cells/cm^2^. After reaching 80% confluency, MSCs were incubated with adipogenic induction medium (α‐MEM supplemented with 1.2 µM dexamethasone, 0.5 mM isobutyl methylxanthine, 100 µM indomethacin, and 10 µg/ml insulin) (Sigma‐Aldrich) for 6 days and then cultured in adipogenic maintenance medium (α‐MEM supplemented with 1.2  µM dexamethasone and 10 µg/ml insulin) for another 8 days, with medium changes every 2 days. MSCs were then fixed with 10% formalin (Sigma‐Aldrich) for 1 h, followed by washing with 60% isopropanol (Fisher Chemical) and incubation with 0.3% Oil Red O solution (Sigma‐Aldrich) for 15 min to stain for lipids.

For osteogenic differentiation, MSCs were seeded into plastic 12‐well plates at 5000 cells cm^−2^. After reaching 80% confluency, MSCs were induced with osteogenic medium (α‐MEM supplemented with 200 nM dexamethasone, 10 mM β‐glycerophosphate, and 50 µM ascorbic acid) (Sigma‐Aldrich) and incubated for 14 days, with medium changes every 3 days. Cells were then fixed with 10% paraformaldehyde and stained with 0.6% Alizarin Red solution (Sigma‐Aldrich) for calcium deposits. Images of lineage‐induced MSCs were captured using the BioTek Cytation 5 widefield microscopy function with 10× magnification and quantified using HALO 4.0 (Area Quantification module) by measuring the area coverage of red staining per image field.

### 4.6 Immunofluorescence Staining, Microscopy, and Quantitative Image Analysis

MSC contractility was assessed using deformable wrinkling silicone substrates, as previously described [139]. MSCs at day 5 were plated on gelatin‐coated (2 µg/cm^2^; G9391, Sigma‐Aldrich) deformable wrinkling substrates. After 24 h, MSC‐generated forces induced the formation of phase‐bright wrinkles, which were captured using a Zeiss Axiovert 135 inverted phase‐contrast microscope equipped with a 20× plan‐apochromat objective (NA 0.60, Ph2) for representative images. For quantification of contraction, MSCs were seeded at ∼20% confluence and recorded with a Cell Discoverer 7 imaging station (Zeiss), with a field diameter of 1.3 mm, allowing for the recording of 15–40 cells per image.

Immunofluorescence staining of cultured cells and tissues was performed as previously described [30]. Briefly, MSCs were fixed with 3% paraformaldehyde in PBS for 10 min, permeabilized with 0.2% Triton X‐100 for 5 min and blocked with 3% BSA in PBS for 1 h. For perilipin‐1 staining, samples were quenched with 250 mM glycine in PBS for 10 min just after fixation. Cells were then incubated for 1 h at room temperature with primary antibodies, including α‐SMA (1:100; SM1, mouse IgG2a), HA‐Tag (1:100; D61E4, rabbit IgG, Cell Signaling Technology), GATA6 (1:100; C29F4, rabbit IgG, Cell Signaling Technology), or perilipin‐1 (1:300; ab3526, rabbit polyclonal, Abcam). After washing threefold for 10 min with PBS, cells were incubated with corresponding TRITC and FITC secondary antibodies (Sigma‐Aldrich), phalloidin–Alexa Fluor 647 (A30107, Invitrogen) to stain F‐actin, and DAPI (32670, Fluka, London, UK) to stain nuclei for 1 h. Fluorescence microscopy images were acquired using an Axio Imager upright microscope equipped with an AxioCam HRm camera, Apotome 2 structured illumination, and ZEN software (Zeiss) with a 20×/0.75 plan‐apochromat objective. Perilipin‐1‐stained samples were imaged using a laser scanning confocal microscope (Zeiss LSM 900) with a Plan‐Apochromat 10×/NA0.75 objective. For every condition, at least five tiled fields with a four‐slice z‐stack were captured.

For tissue staining, paraformaldehyde‐fixed tissue samples were dehydrated through a graded ethanol series and embedded in paraffin. Sections (5 µm thick) were cut using a microtome, deparaffinized in xylene, and rehydrated through a series of ethanol and distilled water baths. For collagen detection, sections were stained using Masson's trichrome according to standard histological protocols. For immunohistochemistry, heat‐induced epitope retrieval was performed in 11.4 mM citrate buffer (pH 6) at 95°C for 20 min, followed by rehydration through three washes with PBS containing 0.1% Tween. Sections were blocked with 10% goat serum for 60 min and incubated overnight with primary antibodies against α‐SMA (1:100; SM1, mouse IgG2a) and CD31 (1:100; ab28364; mouse IgG2a; Abcam). Secondary antibody incubation and nuclear staining were performed as done for cells. Masson's trichrome‐stained sections were scanned using Axio Scan.Z1 microscopy with a Plan‐Apochromat 20×/NA0.8 objective in bright‐field contrast mode. Immunohistochemistry images were acquired with a Zeiss LSM 900 laser scanning confocal microscope using a Plan‐Apochromat 20×/NA0.8 objective, applying Z‐stack and tile functions.

All quantitative image analysis was performed using ImageJ (US National Institutes of Health, Bethesda, MD, USA, http://imagej.nih.gov/ij/, 1997–2013). To measure the percentage of α‐SMA–positive MSCs, images were acquired at 20× magnification at a cell confluence of ∼70%, with every image field containing at least 10–40 cells. Per experimental condition, 10–15 randomly selected images (a total of 100–600 cells) were analyzed per one of at least 3 biological repeats (a total of 300–1800 cells per experimental condition). A previously described ImageJ‐customized macro was applied [30] that uses coherency values to detect α‐SMA–organized stress fibers. The percentage of MFs was determined by dividing the number of α‐SMA–positive cells by the total number of cells, as determined by DAPI staining. For GATA6, the mean fluorescent intensity within the nucleus was assessed using an ImageJ‐customized macro. For adipogenesis analysis, the percentage was calculated as the number of Perilipin‐1–stained cells divided by the total number of cells, as determined by DAPI staining within the image field. For tissue staining, the mean α‐SMA intensity as a measure for MF accumulation was measured at the wound edge in regions lacking CD31 to exclude α‐SMA expressed by vascular smooth muscle cells [30]. Figures were created using Adobe Photoshop C5 (Adobe Systems, San Jose, CA) by enhancing the contrast and brightness consistently for all representative images used. Schematics were designed with BioRender.

For nuclei visualization and CCP analysis, MSCs were fixed with 3% PFA and stained with DAPI [15, 25]. Images were acquired using a laser scanning confocal microscope (Zeiss LSM 900) with a plan‐apochromat 63×/NA1.40 oil‐differential interference contrast objective, at 0.7× zoom for at least 10–20 fields per condition. CCPs were measured based on DAPI intensity within each image field using ImageJ (US National Institutes of Health, Bethesda, MD, USA, http://imagej.nih.gov/ij/, 1997–2013) software, applying the “Find Edges” and “Analyze Particles” algorithms after Gaussian blur processing. CCP values were normalized to the nuclei areas in each image field. CCP and nuclear area measurements were automated using an ImageJ macro script. Wrinkle analysis was performed using ImageJ (US National Institutes of Health, Bethesda, MD, USA, http://imagej.nih.gov/ij/, 1997–2013) by applying a threshold to identify phase‐bright wrinkles and quantifying the surface area of detected particles (wrinkle size: 0–150 µm^2^; circularity: 0.25) in the binary images. Relative contractility was calculated as the wrinkle‐covered area normalized to the number of cells [12].

### 4.7 Animal Experiments and MSC Transplantation

MSC transplantation studies were approved by the Animal Care Committee of St. Michael's Research Vivarium under protocol #340. A total of 16 female Wistar rats (200–250 g) from Charles River Breeding Laboratories were used to generate splinted wound models resembling hypertrophic scars, as previously described [13, 55, 140]. Surgical procedures were performed on a thermally regulated water blanket under Isoflurane inhalation anesthesia. Anesthesia was induced with 3%–5% Isoflurane in a chamber and maintained at 2–2.5% via a nose cone. Each animal received a single subcutaneous injection of the long‐acting analgesic buprenorphine SR (1 mg/kg) immediately before surgery. Eye gel was applied to prevent corneal drying, and dehydration was prevented with a subcutaneous injection of warm saline solution. After shaving the rat's back and disinfecting the area with 70% ethanol and 10% betadine solution, a single 25 × 25 mm full‐thickness open wound, including the cutaneous muscle down to the subcutaneous fascia, was created at shoulder level in the middle of the dorsum using sterile surgical scissors. The wound edges were splinted by a 25 × 25 mm plastic frame using silk sutures (Covidien, SS‐684) to prevent intrinsic wound contraction and induce hypertrophic scarring through mechanical tension.

Freshly trypsinized MSCs (1 million) were mixed with 0.5 mL fibrinogen (50 mg/ml; Sigma‐Aldrich), 0.5 mL thrombin solution (4 U/ml; Sigma‐Aldrich), and 40 µM CaCl_2_ and topically applied to the open splinted wounds. After 2 min, allowing the MSC‐fibrin mixture to polymerize, the wound was covered with a transparent dressing (Tegaderm, 3 M). 9 days post‐wounding, animals were sacrificed using 5% isoflurane followed by intracardiac injection of KCl. Sutures were subsequently removed from the wounds, allowing wound contraction for 15 min, after which wound area reduction was quantified as a measure of wound tension. Wound biopsies were collected from the wound edge, including equal portions of healthy and wounded tissue, fixed overnight in 10% formalin (Sigma‐Aldrich), and further processed for paraffin embedding, sectioning, and staining as described above.

### 4.8 Statistical Analysis

All data are presented as individual values, with statistics calculated as means ± standard deviation from at least three independent biological replicates (MSCs from 3 different rats) unless otherwise noted. Except for the bioinformatic analyses described above, all statistical analyses were performed using GraphPad Prism 10.02. Statistically significant differences between multiple groups were assessed with repeated‐measures one‐way analysis of variance (ANOVA) followed by either Šidák's or Dunnett's post hoc test, while differences between two groups were analyzed with an unpaired *t*‐test. Values with *p *< 0.05 were considered statistically significant.

## Author Contributions

BH secured the primary funding for the study. BH, THB, AEM, and FSY conceived the study plan. BH and FSY wrote the manuscript. RNA‐ and ATAC‐ sequencing preparation was performed in the laboratory ofTHB. FSY performed most experiments. AEM performed ATAC‐seq analysis and BETA analysis. NA, XG, EK, and LD performed specific experiments. All authors contributed to the discussion of the content and wrote sections of the article. All authors reviewed and/or edited the manuscript before submission.

## Funding

The research of BH is supported by a foundation grant (#375597) and Project Grant (#190081) from the Canadian Institutes of Health Research (CIHR) and support from the John Evans Leadership funds (#36050 and #38861) and innovation funds (‘Fibrosis Network, #36349’) from the Canada Foundation for Innovation (CFI) and the Ontario Research Fund (ORF). THB is supported by the National Institute of Health (NIH) funds NIH R01‐HL130918 and NIH R01‐HL155143. AEM was supported by an NIH T32GM136615 grant, and FY received Mary H. Beatty and Harron Fellowships from the University of Toronto.

## Conflicts of Interest

The authors declare no conflict of interest.

## Supporting information




**Supporting File**: advs75226‐sup‐0001‐SuppMat.docx.

## Data Availability

The raw ATAC‐seq and RNA‐seq sequencing data generated in this study have been deposited in the NCBI Gene Expression Omnibus (GEO) and are publicly available under the superseries accession number GSE310578.
