# Supplementary material for: A Circuit of Mechanically Regulated Transcription Factors Balances Regenerative and Fibrotic Memory of Mesenchymal Stromal Cells
Source: Adv Sci (Weinh). 2026 Apr 22:e22056. Online ahead of print. doi: 10.1002/advs.202522056 (PMC13334640; doi:10.1002/advs.202522056)
Supplement: Supplementary file 1 — Supporting File: advs75226‐sup‐0001‐SuppMat.docx. [file ADVS-9999-e22056-s001.docx]

# **Supplementary Materials**


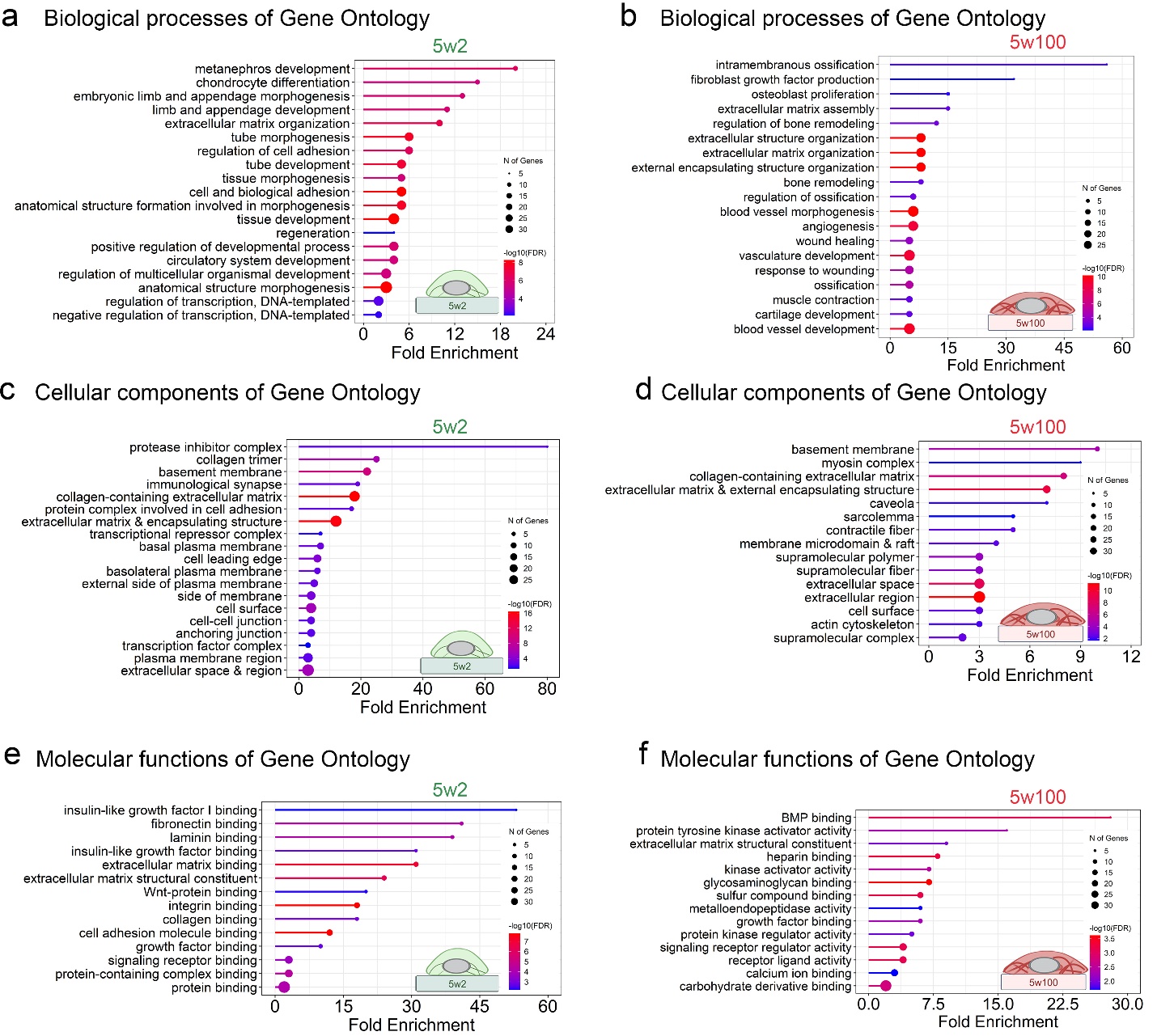


## *Supplementary Figure S1: The role of mechanically induced genes in MSC characterization.*

Gene Ontology (GO) database plots illustrate: (**a, b**) the activity of biological processes, (**c, d**) cellular components, and (**e, f**) molecular functions from the Gene Ontology (GO) database. The enrichment maps highlight the association between functional pathway enrichment and upregulated genes in MSCs primed for 5 weeks on soft surfaces (5w2) (a, c, e) or stiff surfaces (5w100) (b, d, f).


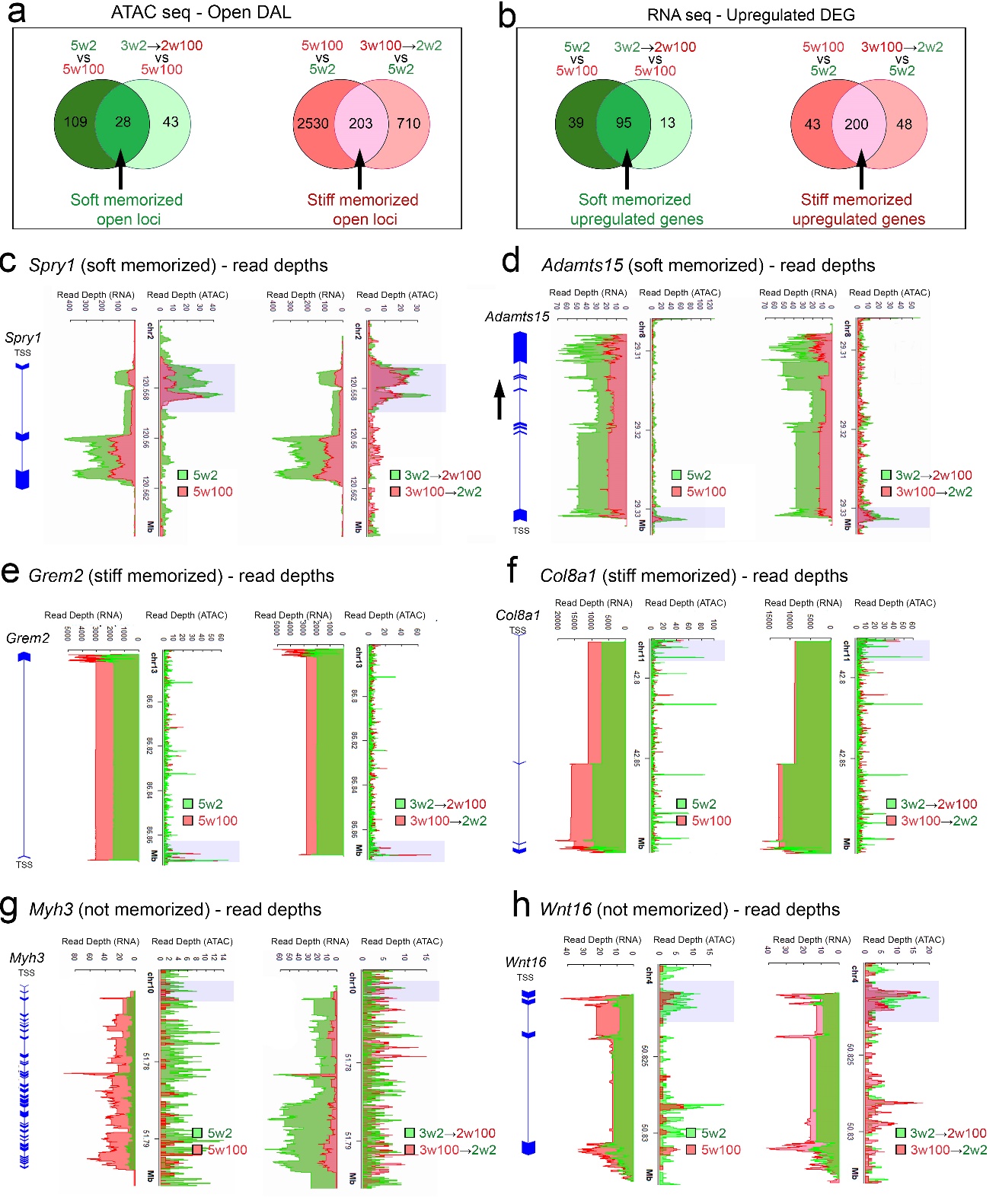


## *Supplementary Figure S2: Gene promoter accessibility states in MSCs with mechanical memory.*

Venn diagrams show (**a**) the number of differentially accessible loci (DAL) (P<0.05) and (**b**) differentially expressed genes (DEG) (P<0.05, log-fold change of >0). Displayed in green are more open DAL and upregulated DEG shared between soft-grown (5w2) and soft-primed, switched MSCs (3w2→2w100), versus (vs) always stiff-grown MSCs (5w100). Displayed in red are more open DAL and upregulated DEG shared between stiff-grown (5w100) and stiff-primed, switched MSCs (3w100→2w2), versus always soft-grown MSCs (5w2). Sushi plots show RNA-seq and ATAC-seq tracks for four selected genes: (**c**) Sprouty RTK signaling antagonist 1 (*Spry1*) and (**d**) ADAM metallopeptidase with thrombospondin type 1 motif 15 (*Adamts15*) exhibit higher accessibility and expression in soft primed and soft-memory MSCs. (**e**) Gremlin 2 (*Grem2*) and (**f**) Collagen type VIII alpha 1 chain (*Col8a1*) are more accessible and expressed in stiff primed MSC and MSC with stiff memory. (**g**) Myosin heavy chain 3 (*Myh3*) and (**h**) *Wnt16* are examples of genes whose expression and accessibility patterns are not preserved by mechanical memory. ATAC-seq and RNA-seq data have been normalized to account for sequencing depth, and the Y-axis scale was chosen to optimize the visualization of peaks for each sample. Green tracks represent soft-primed (5w2) and soft memory MSCs (3w2→2w100); red tracks represent stiff-primed (5w100) and stiff memory MSCs (3w100→2w2). The blue arrows show the direction of genes on the positive strand (downward), and the negative strand (upward), and the purple box indicates the differential accessibility of around promoter regions.


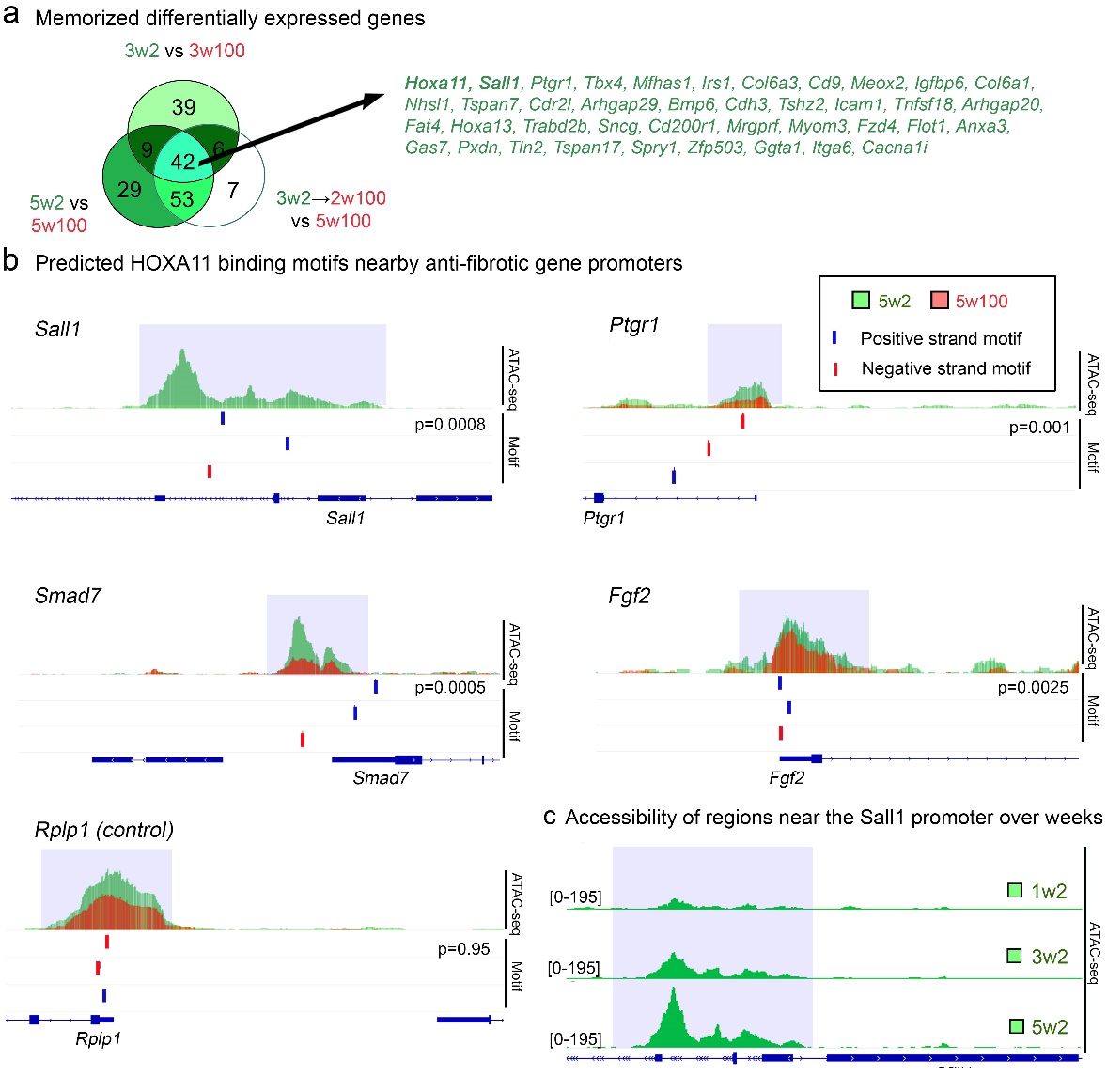


## *Supplementary Figure S3: Predicted HOXA11 binding sites in the promoters of anti-fibrotic genes.*

(**a**) Venn diagram showing differentially expressed genes (DEG) overlapping between soft-primed MSCs at weeks 3 (3w2) and 5 (5w2) (=persistent DEG) and soft-primed MSCs switched to stiff surfaces (3w2→2w100) (=memorized DEG), all significantly upregulated compared to stiff-grown MSCs (P<0.05, log2-fold change>0). (**b**) IN ATAC-seq tracks, HOXA11 binding motifs are indicated as bars in accessible regions near the promoters of *Sall1*, *Ptgr1*, *Smad7*, *Fgf2*, *and Rplp1* (control). Blue bars represent motif position on the positive strand, red bars represent those on the negative strand. Accessible regions were identified from ATAC-seq tracks for soft-primed (green) and stiff-primed (red) MSCs at week 5 (5w2 and 5w100) and are shown in the purple box. The P-value represents the probability of HOXA11 motif occurrence in the respective regions; a lower P-value indicates a more robust prediction of motif presence within the promoter region (**c**) ATAC-seq tracks display promoter accessibility of Sall*1* in soft-primed MSCs over weeks (1w2, 3w2 and 5w2). The ATAC-seq tracks have been normalized to account for sequencing depth, with the Y-axis scales set to the most prominent peak among the 1w2, 3w2, and 5w2 conditions. Blue lines with below each trace indicate whether the gene is located on the positive (right arrows) or negative (left arrows) strand.


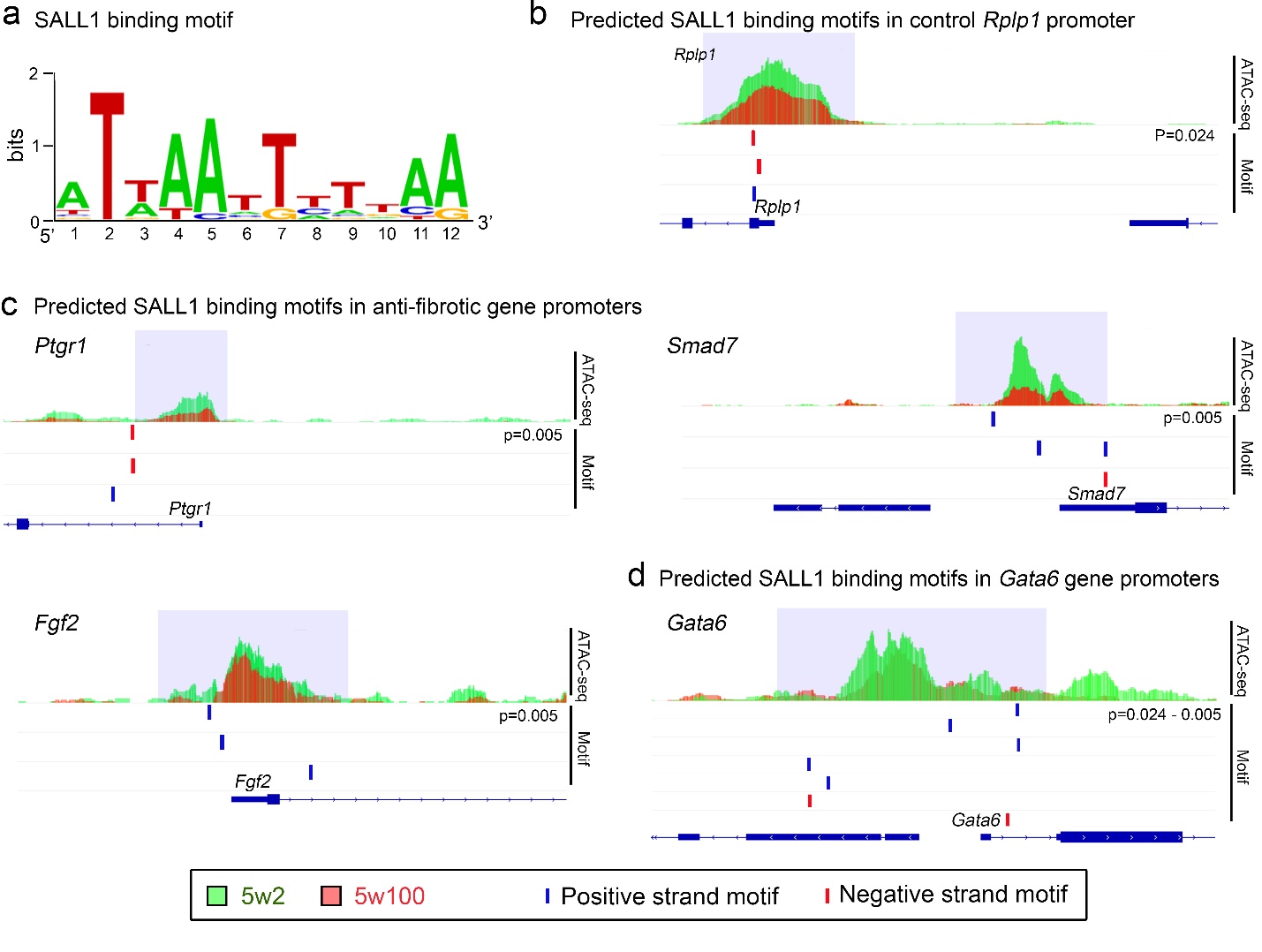


## *Supplementary Figure S4: Predicted SALL1 binding motifs in the promoters of anti-fibrotic genes and Gata6.*

(**a**) Sequence logo representing the SALL1 DNA binding motif, showing nucleotide preferences at each position. Accessible regions in different gene promoters (purple box) were identified from ATAC-seq tracks of 5-week soft-grown (green, 5w2) and stiff-grown (red, 5w100) MSCs. Predicted SALL1 binding motifs are indicated as a bar in accessible regions around (**b**) the *Rplp1* promoter (control) (**c**), the anti-fibrotic genes, *Ptgr1*, *Smad7*, and *Fgf2*, as well as (**d**) *Gata6*. Blue bars represent motif position on the positive strand, and red bars represent those on the negative strand. P-values represent the probability of SALL1 binding motif occurrence in the respective regions; a lower P-value indicates a more robust prediction of motif presence within the promoter region.


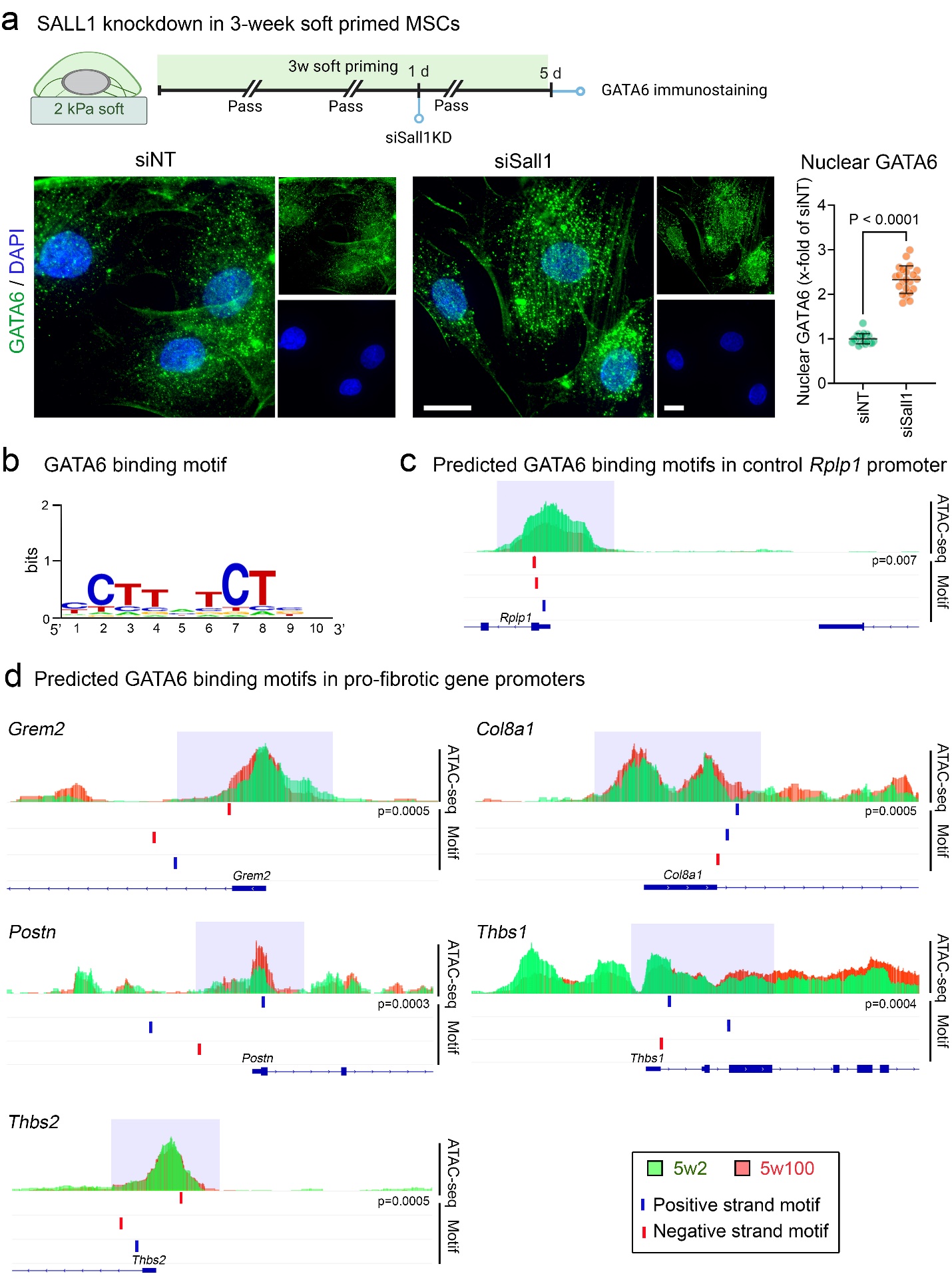


## *Supplementary Figure S5: Effects of SALL1 loss on GATA6 expression and prediction of GATA6 binding motifs in the promoters of pro-fibrotic genes.*

(**a**) SALL1 was knocked down (KD) in 3-week soft-grown MSCs using specific targeting siRNA (siSall1) and non-targeting (siNT) controls. After passing 1 d later plus another 5 d, cells were immunostained for GATA6 (green) and DAPI (blue) to quantify the mean intensity of nuclear GATA6. Scale bars: 20 µm. Column graphs show mean values (±standard deviation) of the experimental repeats. Data points represent one nucleus, with statistics calculated over the averages of all nuclei per experimental repeat. Statistical significance was determined using Student's t-test (n.s., not significant; P<0.05 considered significant). (**b**) Sequence logo representing the GATA6 DNA binding motif, showing nucleotide preferences at each position. Accessible regions in different gene promoters (purple box) were identified from ATAC-seq tracks of 5-week soft-grown (green, 5w2) and stiff-grown (red, 5w100) MSCs. (**c**) Predicted GATA6 binding motifs are indicated as a bar in accessible regions around the promoter of *Rplp1* (control) and (**d**) the pro-fibrotic genes *Col8a1, Postn*, *Grem2, Thbs1*, *and Thbs2.* Promoters with blue bars representing motif position on the positive strand, and red bars representing those on the negative strand. The P-value represents the probability of GATA6 motif occurrence in the respective regions; a lower P-value indicates a higher frequency of motif presence.


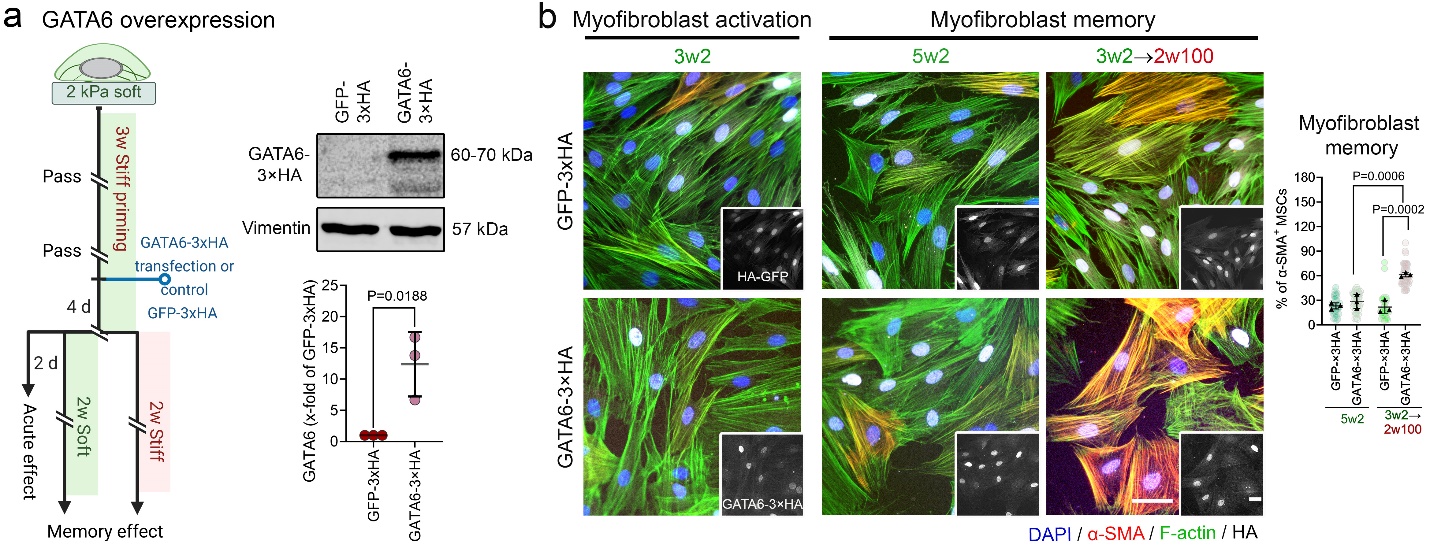


## *Supplementary Figure S6: Overexpression of GATA6 in soft-primed MSCs instills MF memory.*

(**a**) Acute and long-lasting effects of GATA6 overexpression were examined in soft-primed MSCs. MSCs were transfected with a rat GATA6 construct tagged with hemagglutinin (HA) (*Gata6*-3×HA) or control HA-tagged green fluorescent protein (GFP-3xHA) after 3 d in week 3 of soft priming. MSC lysates were collected for Western blot analysis of GATA6 (HA) expression, 4 d days after transfection. (**b**) Acute effects of GATA6 overexpression on MF activation were assessed 3 d after transfection on soft surfaces (3w2) and compared to GFP controls. Memory effects were tested after the switch to stiff surfaces for an additional 2 weeks (3w2→2w100) compared to continued growth on stiff surfaces (5w2). For MF phenotyping, cells were stained for α-smooth muscle actin (α-SMA; red) and F-actin (green), and the % of α-SMA stress fiber–positive MFs was quantified. All scale bars: 100 µm. All column graphs show mean values (±standard deviation, SD) of at least 3 independent biological replicates (N=3). Circular data points in graphs represent a single image field. Black triangles show the mean values calculated across all image fields per experimental repeat, with their standard deviations (SD). Statistical significance was determined for the experimental mean values using Student's t-test or repeated measures analysis of variance (ANOVA) with Šidák's post hoc test (n.s., not significant; P<0.05 considered significant).


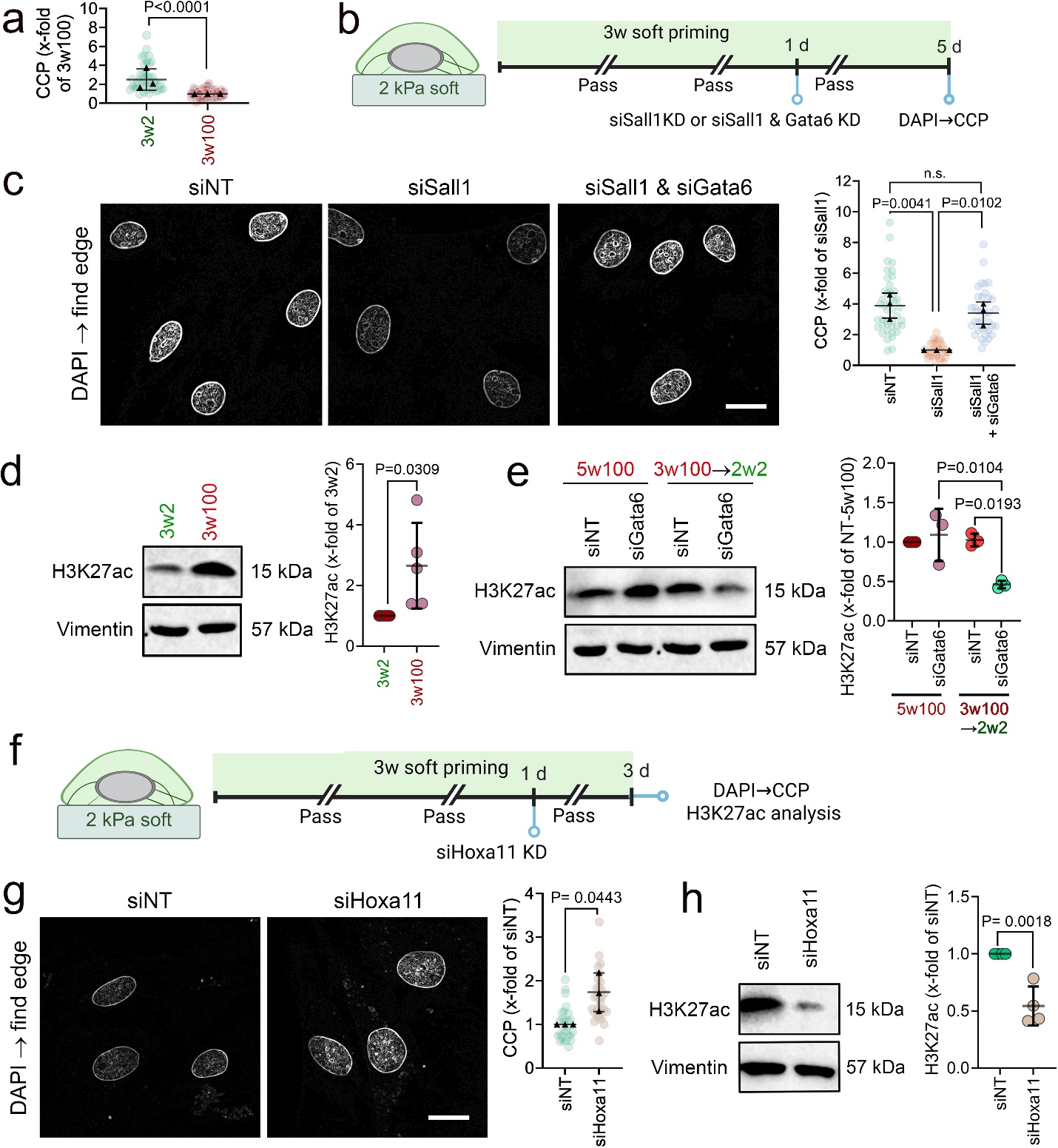


## *Supplementary Figure S7: Manipulation of GATA6 and HOXA11 results in altered chromatin condensation and histone acetylation*

(**a**) Chromatin condensation parameters (CCP) were compared between 3-week soft-primed (3w2) and stiff-primed (3w100) MSCs. CCPs were calculated after applying find-edge image-processing to nuclear DAPI staining. (**b**) SALL1 was knocked down (KD) alone or together with GATA6 in 3-week soft-grown MSCs using gene-specific siRNAs (siSall1, siGata6) and non-targeting control siRNA (siNT). (**c**) One day after transfection, cells were passaged and assessed for CCP after an additional 5 d culture on soft surfaces. (**d**) Lysates from 3w2 and 3w100 MSCs analyzed by Western blotting for H3K27ac. Vimentin was used as a housekeeping control to normalize before calculating the mean H3K27ac signal intensity. Equal relative protein amounts were loaded onto separate gels to account for differences in epitope abundance. (**e**) H3K27ac levels were assessed and quantified by Western blotting in MSCs following GATA6 KD using siGata6 and siNT controls. (**f**) Using the same experimental design, (**g**) HOXA11 was KD in 3-week soft-grown MSCs using siHoxa11 and siNT controls. (**h, i**) CCP (h) and H3K27ac levels (i) were analyzed after 3 d. Scale bar: 20 µm. Independent experiments were performed using MSCs from at least three different rats. All graphs show mean values ± standard deviation (SD) from at least three independent biological replicates (N=3). Circular data points in image-based quantifications represent individual image fields. Black triangles show the mean values calculated across all image fields per experimental repeat, with their standard deviations. Statistical significance was determined for the experimental mean values using repeated-measures analysis of variance (ANOVA) with Šidák’s post hoc test, except for panel (b), which was analyzed using Dunnett’s test (n.s., not significant; P < 0.05 considered significant).
